# *HvSL1* and *HvMADS16* promote stamen identity to restrict multiple ovary formation in barley

**DOI:** 10.1093/jxb/erad218

**Published:** 2023-06-03

**Authors:** Caterina Selva, Xiujuan Yang, Neil J Shirley, Ryan Whitford, Ute Baumann, Matthew R Tucker

**Affiliations:** Waite Research Institute, School of Agriculture Food and Wine, University of Adelaide, Waite Campus, Urrbrae 5064, South Australia, Australia; Waite Research Institute, School of Agriculture Food and Wine, University of Adelaide, Waite Campus, Urrbrae 5064, South Australia, Australia; Waite Research Institute, School of Agriculture Food and Wine, University of Adelaide, Waite Campus, Urrbrae 5064, South Australia, Australia; Waite Research Institute, School of Agriculture Food and Wine, University of Adelaide, Waite Campus, Urrbrae 5064, South Australia, Australia; Waite Research Institute, School of Agriculture Food and Wine, University of Adelaide, Waite Campus, Urrbrae 5064, South Australia, Australia; Waite Research Institute, School of Agriculture Food and Wine, University of Adelaide, Waite Campus, Urrbrae 5064, South Australia, Australia; University of Massachusetts Amherst, USA

**Keywords:** Barley, B-class, cereal, C2H2, flower development, MADS-box, multiovary, transcription factors, *Triticeae*

## Abstract

Correct floral development is the result of a sophisticated balance of molecular cues. Floral mutants provide insight into the main genetic determinants that integrate these cues, as well as providing opportunities to assess functional variation across species. In this study, we characterize the barley (*Hordeum vulgare*) multiovary mutants *mov2.g* and *mov1*, and propose causative gene sequences: a C2H2 zinc-finger gene *HvSL1* and a B-class gene *HvMADS16*, respectively. In the absence of *HvSL1*, florets lack stamens but exhibit functional supernumerary carpels, resulting in multiple grains per floret. Deletion of *HvMADS16* in *mov1* causes homeotic conversion of lodicules and stamens into bract-like organs and carpels that contain non-functional ovules. Based on developmental, genetic, and molecular data, we propose a model by which stamen specification in barley is defined by *HvSL1* acting upstream of *HvMADS16*. The present work identifies strong conservation of stamen formation pathways with other cereals, but also reveals intriguing species-specific differences. The findings lay the foundation for a better understanding of floral architecture in *Triticeae*, a key target for crop improvement.

## Teaser

Analysis of barley multiovary *mov1*and *mov2*loci indicates that HvSL1 and HvMADS16 exhibit both unique and conserved roles in the specification and development of cereal florets.

## Introduction

The ABC model of flower development postulates that each organ within a flower is specified by the combinatorial action of distinct gene classes, referred to as the A-, B-, C-, D-, and E-class genes (Schwarz-Sommer *et al*., 1990; Coen and Meyerowitz, 1991; Colombo *et al*., 1995). These genes act in concentric rings (whorls) of tissue within the floral meristem to establish organ identity. Generally, A+E-class genes control the development of sepals (or bract-like organs) in the outermost whorl (whorl 1), while the action of A+B+E-class genes in whorl 2 gives rise to petals or equivalent structures. Stamens in the third whorl are specified by B+C+E-class genes while, in the fourth innermost whorl, carpels require C+E-class genes, and D-class genes are essential for ovule development. Most genes within the ABC model encode transcription factors belonging to the MADS-box gene family (Coen and Meyerowitz, 1991; Pelaz *et al*., 2000; Favaro *et al*., 2003). Numerous studies have added to this model over time, creating a more comprehensive view of the complex genetic network leading to flower formation (Thomson and Wellmer, 2019). Importantly, dimers of MADS-box proteins can interact to form tetrameric complexes, described as the floral quartet model (Theissen, 2001). The floral tetramers can promote DNA looping to bring distal promoter regions closer and allow recruitment of transcription cofactors, chromatin-remodelling proteins, and other transcription factors to regulate the expression of downstream genes (Melzer and Theissen, 2009; Smaczniak *et al*., 2012).

Cys_2_/His_2_ (C2H2) zinc-finger transcription factors also play an important role in flower development by acting upstream and downstream of MADS-box genes, and by coordinating cell proliferation and differentiation during floral organogenesis (Lyu and Cao, 2018). For example in Arabidopsis, *JAGGED* (*JAG*) functions during petal primordia formation and redundantly acts with its paralogue *NUBBIN* (*NUB*) to control lateral growth and differentiation of stamens and carpels (Dinneny *et al*., 2004, 2006). In rice (*Oryza sativa*), the *JAG* orthologue *STAMENLESS1* (*SL1*) specifies lodicule and stamen identity (Xiao *et al*., 2009).

Understanding floral development provides opportunities to manipulate floral structures for application in agriculture. In crop species, considerable effort has been devoted to understanding flower development in rice (Arora *et al*., 2007; Yoshida and Nagato, 2011) and maize (*Zea mays*) (Bommert *et al*., 2005; Bartlett *et al*., 2015; Ali *et al*., 2019; Abraham-Juárez *et al*., 2020). Less information is available for members of the *Triticeae*, such as wheat (*Triticum aestivum*) and barley (*Hordeum vulgare*), despite their importance in the food and feed industries. Recent functional studies (e.g. on *HvMADS1*, *HvLFY*, *HvAP2*, and *HvMADS29*; Li *et al*., 2021; Selva *et al*., 2021; Shoesmith *et al*., 2021), coupled with mining of genomic resources have started to address the diversity of ABC genes in barley (Callens *et al*., 2018; Kuijer *et al*., 2021). This has important implications for the *Triticeae* cereals, where manipulating floral structures to improve hybrid grain production is still a promising avenue for increased yield and crop improvement (Selva *et al*., 2020).

Diversity in floral structure can be obtained through specific targeting of regulatory genes (e.g. CRISPR/Cas9; Li *et al*., 2021; Selva *et al*., 2021), through the exploitation of natural genetic variation (e.g. in barley; Caldwell *et al*., 2004; Talamè *et al*., 2008; Druka *et al*., 2011; Szarejko *et al*., 2017; Szurman-Zubrzycka *et al*., 2018; Schreiber *et al*., 2019; and wheat; Krasileva *et al*., 2017), or through the induction of mutations by chemical mutagenesis (Knudsen *et al*., 2022). In terms of the latter, historical forward genetic screens have uncovered an array of floral phenotypes. Of these, pistillody is the conversion of stamens into additional pistils (Murai and Tsunewaki, 1993; Murai *et al*., 2002; Peng, 2003). Pistillody is one avenue to increase the number of seed-bearing units per plant (Yang and Tucker, 2021), as well as to create a male-sterile mother plant for cross-pollination in hybrid breeding (Selva *et al*., 2020). Studies in wheat mutants often show that pistillody correlates with changes in expression of genes belonging to the ABC model (Yamada *et al*., 2009; Wang *et al*., 2015; Yang *et al*., 2015). This is particularly true for B-, C-, and D-classes, which are directly implicated in stamen, carpel, and ovule development.

Until now, at least three pistillody loci have been reported in barley: *multiovary1* (*mov1*), *mov2*, and *mov5* (Franckowiak and Lundqvist, 2013). Early linkage and mapping attempts positioned the *mov1* locus, proposed to encode a MADS-box gene, on chromosome 7H (Tazhin, 1980; Soule *et al*., 1996, 2000). The *mov2* locus was mapped to chromosome 3H and also suggested to encode a member of the MADS-box gene family (Soule *et al*., 1996, 2000). The barley *mov5* locus was recently characterized and shown to encode a FLORICAULA/LEAFY (HvLFY) transcription factor on chromosome 2H (Selva *et al*., 2021). Despite a general description of the mutant phenotype and rough mapping of the *mov2* and *mov1* loci, the causative gene sequences have yet to be reported.

To increase our knowledge of floral development in the *Triticeae*, this study aimed to map and characterize the barley *mov1* and *mov2* loci. We show that *mov2* maps to a region on 3H encoding a C2H2 zinc-finger transcription factor, named here as HvSL1 based on homology to rice STAMENLESS1 (Xiao *et al*., 2009). Moreover, we show that a total of three genes on chromosome 7H, including the MADS-box B-class gene *HvMADS16*, are absent in *mov1* plants. We investigate the interaction of HvSL1 with barley B-class genes, report the developmental changes and effect on ABC gene expression during floral development in *mov1* and *mov2.g*, and propose a model for stamen specification in barley. Our results highlight strong conservation in the core determinants of stamen formation, despite intriguing differences in MADS-box regulation between cereals.

## Materials and methods

### Plant material and genotyping

Grains segregating for the *mov2.g* allele (*mov2* locus) and for the *mo6b* allele (*mov1* locus) in cv. Steptoe were kindly provided by Professor A. Kleinhofs. Germination was synchronized by cold treatment at 4 °C for 3 d in the dark. The germinated grains were then transplanted into pots and grown in the glasshouse at 23 °C/16 °C day/night temperatures and long days (~12 h). Phenotyping was done at the heading stage by visually inspecting the central florets of several spikes per plant. Florets were imaged with a Nikon SMZ25 Stereo Fluorescence Microscope equipped with DS-Ri1 colour-cooled digital camera.

Genotyping of the plants was performed by copy number analysis using TaqMan™ assay (Thermo Fisher Scientific, USA) designed for *HvSL1* and *HvMADS16* as genes of interest. The barley *CONSTANS*-like *CO2* (*HORVU6Hr1G072620*) was used as the internal positive control (Kang *et al*., 1997). The sequences of primers and probes can be found in Supplementary Table S1. The reaction was set up as follows: 1× PrecisionFast™ qRT-PCR Master Mix with Low ROX (Primerdesign Ltd, UK), 200 nM of each primer, 100 nM of probe, 150 ng of template DNA, and water to a final volume of 10 μl. The reaction was performed with an initial activation step at 95 °C for 2 min, followed by 40 cycles at: 95 °C for 5 s and 60 °C for 20 s in a QuantStudio 6 Flex Real-Time PCR machine (Thermo Fisher Scientific, USA). The detectors used were FAM-BHQ1 and HEX-BHQ1, with ROX as internal passive reference.

The *mov2* bi-parental mapping population was created by manually cross-pollinating *mov2.g* florets (cv. Steptoe) with wild-type pollen from cv. Morex. Heterozygosity of F_1_ plants was confirmed by KASP™ marker analysis across two known single nucleotide polymorphisms (SNPs), at positions chr3H_1006543 and chr3H_28805649, and by Sanger sequencing across a third SNP at position chr7H_557244517 according to the Morex reference assembly (Hv_IBSC_PGSB_v2). For fine-mapping of the *mov2* locus, SNPs spanning the 3HS region were identified and developed as KASP™ markers. SNPs were identified from a published Steptoe×Morex dataset (Close *et al*., 2009) and from Steptoe leaf transcriptomic data mapped to the reference Hv_IBSC_PGSB_v2 Morex assembly. Primer Picker (LGC Genomics) and the LGC Genomics SNPline™ were used to design and prepare the KASP™ marker assays. Assays were performed using KASP™ Master mix as instructed by the manufacturer. Sequences of KASP™ markers can be found in Supplementary Table S2.

### Nucleic acid extraction and genomic PCR

For genotyping, genomic DNA was extracted from freeze-dried 2-week-old plant material using a phenol–chloroform method as described by Rogowsky *et al.* (1991). PCR was performed using Q5® high-fidelity DNA polymerase (New England BioLabs, USA), following the manufacturer’s protocol in a final volume of 12.5 μl. All PCR products were separated by agarose gel electrophoresis, purified using the ISOLATE II PCR and Gel Kit (Bioline, Australia), and Sanger sequenced at the Australian Genome Resource Facility (AGRF). Primers used for PCR can be found in Supplementary Tables S3–S5.

### RNA extraction, cDNA synthesis, and quantitative real-time PCR (qRT-PCR)

Ultra fine-pointed tweezers were used to manually dissect inflorescences at Waddington (W) developmental stages W2.0, W3.5, W4.0, and W6.0 (Waddington *et al*., 1983), corresponding to ~17, 20, 23, and 26 d post-germination in the growing conditions described above. Inflorescences were snap-frozen in liquid nitrogen, RNA extraction was performed using the ISOLATE II RNA Plant Kit (Bioline, Australia), followed by TURBO™ DNase treatment (Thermo Fisher Scientific, USA), and cDNA synthesis using the SuperScript™ IV First Strand Synthesis (Thermo Fisher Scientific, USA) as per the manufacturers’ instructions.

RNA from transfected protoplasts was extracted using a phenol–chloroform method. Briefly, samples were lysed in a 1.5 ml tube and homogenized in 500 μl of TRIzol (Thermo Fisher Scientific, USA) before adding 150 μl of chloroform. Samples were vortexed and centrifuged at maximum speed at 4 °C for 15 min. The aqueous phase was carefully transferred to a new 1.5 ml tube with 250 μl of isopropanol and incubated at 4 °C for 10 min before centrifuging at maximum speed at 4 °C for 20 min. The precipitated RNA was washed with 500 μl of 100% ethanol, allowed to air-dry, and resuspended in 20 μl of diethylpyrocarbonate (DEPC)-treated water.

qRT-PCR was carried out as described in Selva *et al*. (2021). Each time point is the result of three technical replicates and at least three biological replicates. Absolute quantification is reported as normalized transcript against the geometric means of the three least varying control genes as described in Vandesompele *et al*. (2002). Relative quantification in transfected protoplasts is expressed as calibrated normalized relative quantities (NRQ) as calculated by the qbase+ software (Biogazelle, Belgium; Hellemans *et al*., 2007). Primer information for the reference genes and genes of interest can be found in Supplementary Table S6.

### CRISPR and plant transformation

Guide RNA (gRNA) design and cloning for *HvSL1* CRISPR (clustered regularly interspaced palindromic repeats)-associated protein knockout was done following the procedure described by Ma *et al.* (2015) with vectors kindly provided by Professor Yao-Guang Liu (South China Agricultural University). Two gRNAs targeting positions +42 bp and +275 bp from the translational start site of *HvSL1* were cloned in the same vector. Primer sequences used for cloning are listed in Supplementary Table S7.

*Agrobacterium tumefaciens*-mediated plant transformation (cv. Golden Promise) and genotyping of regenerant plants was performed as described previously (Selva *et al*., 2021).

### *In situ* hybridization

Sense and antisense RNA probes for *in situ* hybridization were amplified using Q5® high-fidelity DNA polymerase (New England BioLabs, USA) with the T7 promoter extension to the 5ʹ end of primers (primers used can be found in Supplementary Table S8), transcribed, and digoxigenin (DIG) labelled. Inflorescences were prepared and *in situ* hybridization was performed as described in Selva *et al*. (2021)

### Bi-molecular fluorescent complementation (BiFC)

The full-length coding sequences of *HvMADS2*, *HvMADS4*, *HvMADS16*, and *ΔHvMADS16*, containing only the MADS-domain (amino acids 1–65), were PCR-amplified with Q5® high-fidelity DNA polymerase (New England BioLabs, USA) and cloned in the BiFC vectors pSAT1-nEYFP-N1 (N-terminal fragment) and pSAT1-cEYFP-C1-B (C-terminal fragment) (Citovsky *et al*., 2006). Primers used for cloning are listed in Supplementary Table S9. A cyan fluorescent protein (CFP) co-transformation control and positive interaction control (OsERS1–nYFP and OsARC–cYFP) were included (Yang *et al*., 2018). All plasmids were checked by digestion and Sanger sequencing. Biolistic particle bombardment of onion epidermal cells was carried out as described in Selva *et al*. (2021). For each interaction test, four independent transfections were undertaken, including two technical replicates and at least two biological replicates. Interaction frequency was similar for each combination, showing a positive result, and was comparable with the positive control.

### Light and electron microscopy

For light microscopy, wild-type, *mov2*, and *mov1* carpels were fixed in FAA solution (50% ethanol, 5% glacial acetic acid, 10% formaldehyde, one drop of Tween-20) overnight, dehydrated in a 70–100% ethanol series, and embedded in LR white resin. Samples were sectioned using a Leica Rotary Microtome RM2265 at 1.5 μm. Slides were stained with 0.1% toluidine blue in 0.1% sodium tetraborate for 2 min and rinsed three times with water, dried, and mounted with DPX. After 72 h, slides were imaged with a Nikon Eclipse Ni-E optical microscope equipped with a DS-Ri1 colour cooled digital camera. Image analysis and processing were carried out with the NIS-Elements AR software.

For SEM, inflorescences were manually dissected and fixed overnight in 4% paraformaldehyde, 1.25% glutaraldehyde in phosphate-buffered saline (PBS), 4% sucrose, pH 7.2. Before processing, samples were washed three times in PBS and fixed in 2% OsO_4_ in PBS for 1 h. Samples were then dehydrated in a 50–100% ethanol series and dried with a critical point dryer. Dried samples were arranged on carbon tabs stuck to 12 mm aluminium stubs and coated with platinum. Samples were observed using a Hitachi FlexSem 1000 scanning electron microscope.

### Protoplast isolation

Isolation of barley leaf protoplasts was performed as described by Yoo *et al*. (2007) with minor modifications. Briefly, the adaxial epidermal layer of leaves from 11-day-old barley seedlings (cv. Golden Promise) was manually peeled off and the leaf was cut into ~2 cm×0.5 cm strips using surgical blades. The leaf segments of ~10 plants were immediately transferred to a Petri dish containing 15 ml of 0.6 M mannitol for 30 min at room temperature to induce plasmolysis. After incubation, the leaf segments were transferred to another Petri dish containing 10 ml of freshly prepared enzyme solution [0.55 M mannitol, 40 mM MES-KOH at pH 5.7, 20 mM KCl, 2.0% cellulase R10 (Yakult Pharmaceutical, Japan), 0.75% macerozyme R10 (Yakult Pharmaceutical), 10 mM CaCl_2_, 0.1% BSA] and incubated for 3 h in the dark at 28 °C with gentle shaking (40–60 rpm). After enzymatic digestion, forceps were used to gently remove the remaining epidermis and leaf debris from the enzyme solution. An equal volume (10 ml) of W5 solution (154 mM NaCl, 125 mM CaCl_2_, 5 mM KCl, and 2 mM MES-KOH at pH 5.7) was slowly added to the protoplasts and the solution was filtered with a 100 μM nylon mesh into a 50 ml round-bottom tube. The volume was adjusted by adding 5 ml of W5 solution. The filtered protoplasts were collected by centrifugation at 600 *g* for 3 min. The supernatant was replaced with 15 ml of fresh W5 and the protoplasts resuspended by gentle shaking. Protoplasts were allowed to pellet by gravity for 30 min in ice. After incubation, the supernatant was promptly removed and substituted with MMG solution (0.6 M mannitol, 15 mM MgCl_2_, and 4 mM MES-KOH at pH 5.7) at a concentration of 10^6^ cells ml^–1^, determined by counting cells in 12 μl of a 1:10 dilution of protoplast solution with a haematocytometer.

### PEG-mediated transfection of barley protoplasts

Polyethylene glycol (PEG)-mediated transfections were mostly carried out as described by Bai *et al*. (2014). Firstly, 200 μl of PEG–Ca^2+^ solution [40% (w/v) PEG 4000, 0.4 M mannitol, 0.1 M CaCl_2_] were pre-loaded in pipette tips. Secondly, 100 μl of protoplast solution (~10^5^ cells) were added to 20 μg of each plasmid DNA in a 2.0 ml tube. The pre-loaded PEG–Ca^2+^ solution was immediately added to the protoplast–DNA mixture, mixed gently, and incubated for 15 min at room temperature in the dark. The transfection process was stopped by adding 840 μl of W5 and centrifuged at 600 *g* for 2 min. Cells were resuspended in 500 μlof W5 and transferred to multi-well plates previously coated with 5% (v/v) foetal bovine serum (FBS). Protoplasts were cultured at 28 °C for 40–48 h in the dark.

To test transfection efficiency, protoplasts were transfected with pUbi-YFP-rbcS in six independent transfections. At 40–48 h after transfection, the protoplasts were visualized under UV and bright light using a Nikon Eclipse Ni-E optical microscope equipped with a DS-Ri1 colour cooled digital camera.

## Results

### *mov2.g* florets have functional supernumerary carpels and produce multiple grains

**Fig. 1.**
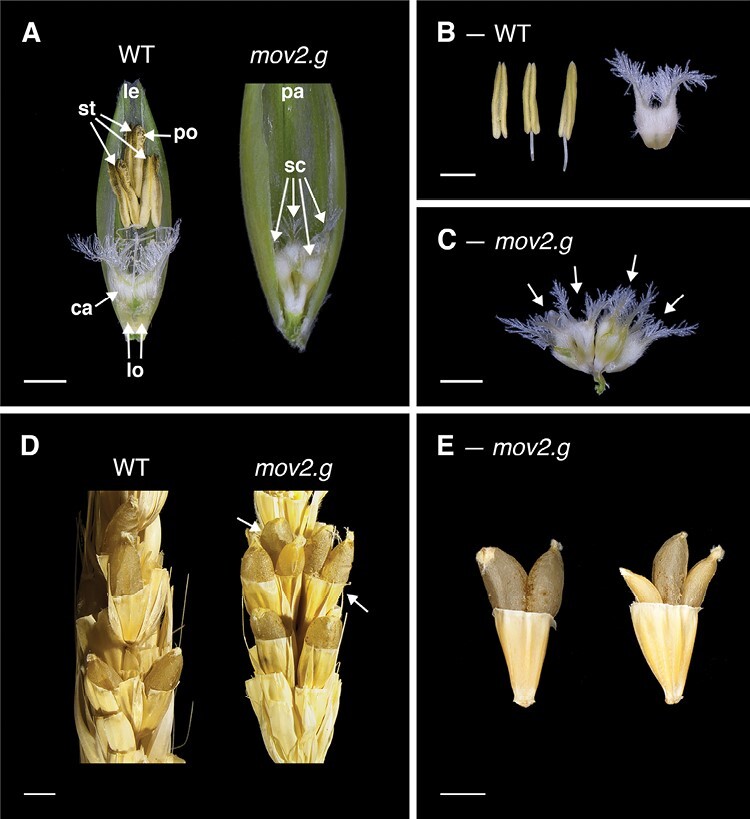
Florets, reproductive organs, and grains in the wild type (WT) and *mov2.g*. (A) Exposed WT and *mov2.g* florets. Palea or lemma have been removed to show internal floral organs. (B) Reproductive organs in WT florets consist of three stamens and one carpel. (C) Reproductive organs in *mov2.g* florets consist of supernumerary carpel-like structures (arrows). Scale bars: 1000 µm. (D) Manually cross-pollinated WT and *mov2.g* spikes. White arrows indicate multiple grains per floret. (E) Examples of multiple grains per floret produced by manually cross-pollinated *mov2.g* spikes. Scale bars: 2000 µm. Lemma (le), palea (pa), lodicule (lo), stamen (st), carpel (ca), supernumerary carpel-like structure (sc), pollen (po).

In wild-type barley, floral organs are arranged in a defined pattern such that the outermost whorl contains the palea and lemma (whorl 1). These are followed by two lodicules in whorl 2 and three stamens in whorl 3, surrounding a single carpel at the centre of the floret in whorl 4 (Fig. 1A, B) (Brenchley, 1920).

In florets from *mov2.g* plants, the stamens are replaced by supernumerary carpels, rendering the plant unable to self-pollinate (Fig. 1A). The number of carpels within each floret typically varies between four and seven carpels or carpel-like structures (Supplementary Table S10). Visually, all carpels appear irregularly shaped, connected at the base, and of smaller size relative to a wild-type carpel (Fig. 1C). In contrast, development and morphology of the lemma, palea, and lodicules remains largely unaffected. Most notably, when *mov2.g* plants were used as a female recipient in manual cross-pollination, most florets were able to produce multiple grains with a maximum of three developing grains within a single floret (Fig. 1D, E). This is due to the ability of some *mov2.g* carpel structures to correctly differentiate fully functional female gametophytes, as was observed in transverse sections stained with toluidine blue (Supplementary Fig. S1). All grains from *mov2.g* plants were viable, and after germination gave rise to mature plants.

**Fig. 2.**
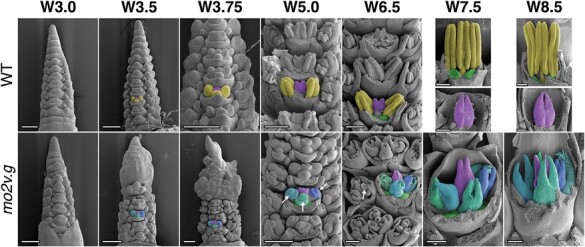
SEM of *mov2.g* developing inflorescences. In the wild type (WT), primordia giving rise to stamens are false-coloured in yellow, cells giving rise to the carpel in purple, and lodicules in green. In each panel from W3.5 onwards, the primordia are false-coloured in a single floret to enable comparison between genotypes. In *mov2.g*, the central carpel (purple) and lodicules (green) appear to be retained; cells giving rise to the additional carpel-like structures are false-coloured in blue (different shades). White arrows indicate ovule primordia. Waddington stage is indicated for each developmental time point; from stage W5.0, lemma and/or stamens have been removed to expose the carpels. Scale bars: 200 µm.

To better understand the developmental basis of the multiovary phenotype, SEM was used to compare wild-type (cv. Steptoe) and *mov2.g* inflorescences. Immature inflorescences were morphologically similar at the very early stages of development [i.e. floret primordium, at W3.0; Fig. 2; Waddington *et al*., 1983]. At stages W3.5–W3.75, both wild-type and *mov2.g* floral meristems initiated lateral dome-shaped protrusions, consistent with the appearance of stamen primordia from the floral meristem. Following this stage, the first morphological differences were identified. In the wild type, the stamen primordia differentiated into filament and anther tissues (W5.0–W8.5), while the meristematic tissue at the centre proliferated and terminally differentiated into a single ovule-containing carpel. As each wild-type floral meristem developed, a vertical symmetry along the central inflorescence rachis was maintained.

In contrast, in *mov2.g*, the lateral protrusions did not differentiate into stamens, but instead gave rise to organs that followed the characteristic morphogenesis of carpels (W5.0–W8.5) (Fig. 2). Interestingly, until W5.0, only three organ primordia were present in the third whorl, probably originating from converted stamen primordia. However, the number of carpels in whorl 3 dramatically increased by W6.5 and appeared to develop synchronously with the central carpel. These carpel-like organs initially developed an ovule primordium that was later surrounded by the growing carpel tissue. By stage W8.5, the ectopic carpels also differentiated stigmas bearing stigmatic papillae. Moreover, as floral organs developed, there was frequent loss in the vertical symmetry of florets along the central inflorescence rachis, and occasional fasciation of the growing tip, which was then reflected in a shorter and broader spike compared with the wild type (Supplementary Fig. S2). The inflorescence fasciation trait, although interesting, was not investigated further here.

### *mov2* encodes a C2H2 zinc-finger transcription factor

A previous study had mapped the barley *mov2* locus to the telomeric region on the short arm of chromosome 3H (3HS) within an ~28 Mb genomic interval (Soule *et al*., 2000). This interval is too large to reliably identify the underlying causative gene(s). Therefore, a *mov2.g* (cv. Steptoe)×Morex bi-parental population was developed to map *mov2* to a higher genetic resolution. The multiovary phenotype was not observed in F_1_ individuals, indicative of the *mov2.g* mutation being completely recessive. A total of 352 F_2_ plants were grown until maturity and used for recombination-based genetic mapping.

Seven KASP™ markers designed to span the previously published interval confirmed that *mov2* mapped to the expected region on chromosome 3H. Following this, 10 additional KASP™ markers were designed to saturate the interval, reducing the critical *mov2*-containing region to ~1.9 Mb, based on flanking markers chr3H_9095799 and chr3H_11039299 (Supplementary Fig. S3). Further resolution of the locus was achieved with six additional KASP™ markers in 179 F_3_ individuals segregating for the multiovary phenotype. Comparison of phenotypic and genotypic segregation across these F_3_ plants reduced the critical interval to ~449 kb, between markers chr3H_9748112 and chr3H_10289104 (Supplementary Fig. S3). This region contains 20 annotated gene sequences, based on the Morex genome reference sequence (Supplementary Table S11).

Expression in floral tissues was assessed for each of the 20 annotated gene sequences to identify the likely causative gene, especially in tissue types affected in *mov2.g* florets. Barley expression datasets were obtained from a range of transcriptomes including 2-week-old seedlings (Zadoks stage Z12; Liu *et al*., 2019), developing inflorescences at stages W2.0, W3.5, and W8.0–W8.5 (Liu *et al*., 2019), developing pistils (W8.0–W10.0; Matros *et al*., 2021), and wild-type (cv. Steptoe) and *mov2.g* leaves (Z22; Zadoks *et al*., 1974). Overall, 10 of the 20 annotated gene sequences showed expression in either pistils or inflorescences. Of these, only *HORVU3Hr1G003740* was identified to be uniquely expressed in reproductive tissues but not in vegetative tissues (leaf and seedling stage) or in *mov2.g* leaves (Supplementary Fig. S4). Sequence analysis indicated that *HORVU3Hr1G003740* encodes a putative C2H2 zinc-finger transcription factor sharing 65.4% sequence identity with the rice protein STAMENLESS1 (OsSL1) (Supplementary Fig. S5; Supplementary Tables S12, S13). In rice, *OsSL1* is known to play a crucial role in floral development, with loss of *OsSL1* function leading to a multiovary phenotype (Xiao *et al*., 2009). Thus, *HORVU3Hr1G003740* is hereafter referred to as *HvSL1*.

**Fig. 3.**
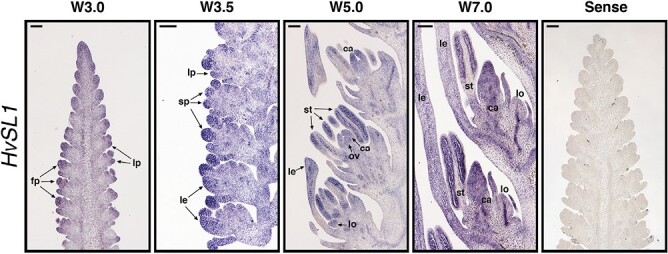
Spatial expression of *HvSL1* during wild-type inflorescence development. Waddington stages correspond to lemma–floret primordia (W3.0), stamen primordia (W3.5), ovule primordium (W5.0), and stamen and carpel development (W7.0). Annotations and arrows indicate floral primordia (fp), lemma primordia (lp), stamen primordia (sp), lemma (le), lodicule (lo), stamen (st), carpel (ca), and ovule (ov). A sense *HvSL1* probe was used as negative control to determine probe specificity. Scale bars: 100 µm.

To validate the transcriptomic data, mRNA *in situ* hybridization was performed to confirm the *HvSL1* spatial expression pattern in wild-type developing inflorescences. At stage W3.0, *HvSL1* expression accumulated in the floret primordia, including the inflorescence tip, and was progressively found in the primordia of glumes, lemma, lodicules, stamens, carpels, and ovules (W3.5–W5.0) (Fig. 3). As the floral organs developed (W7.0), the signal persisted in the stamens, in the carpel, at the apical tip, and in the central vasculature of the lemma, but had decreased in the lodicules.

### *HvSL1* is deleted in *mov2.g* plants and its absence causes multiovary

To determine whether the *mov2.g* phenotype is a consequence of mutation in *HvSL1*, PCR primers were designed to amplify the entire *HvSL1* coding sequence. Comparisons between Morex, Steptoe, and *mov2.g* (cv. Steptoe) genomic DNA suggested that *HvSL1* is absent in *mov2.g* plants but present in both Steptoe and Morex (Supplementary Fig. S6A). In contrast, predicted high-confidence neighbouring gene sequences were still present, indicating that *HvSL1* could be the only deleted gene at the *mov2* locus (Supplementary Table S11). For additional confirmation, the *HvSL1*-specific PCR was repeated on 36 critical recombinant F_3_ individuals from the *mov2.g*×Morex mapping population. For all samples, the absence of *HvSL1* correlated with mutant phenotype expression. Furthermore, *HvSL1* expression by qRT-PCR in developing inflorescences indicated that *HvSL1* expression was completely absent in *mov2.g* samples (Supplementary Fig. S6B), whereas *HvSL1* transcript abundance increased in the wild type between stages W2.0 and W6.0 (double ridge—carpel development), with the biggest increase between W4.5 (carpel primordium) and W6.0.

**Fig. 4.**
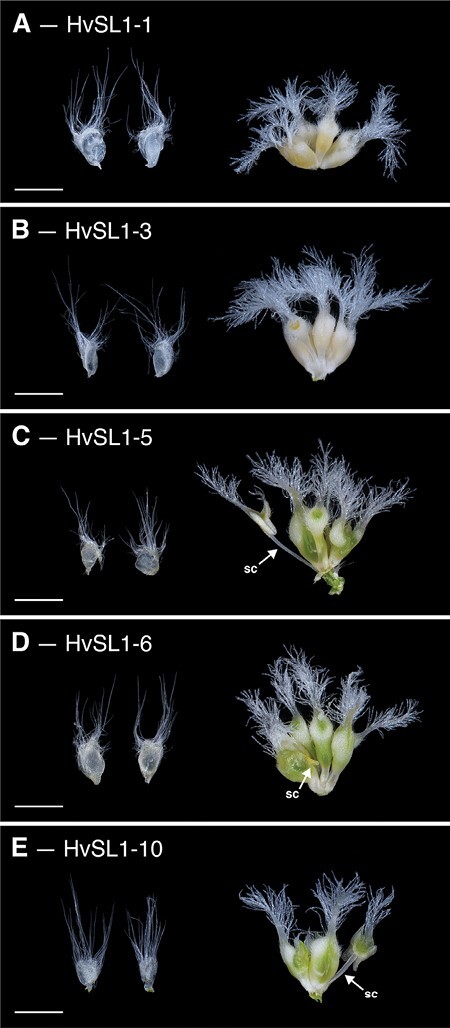
Phenotype of CRISPR *Hvsl1* knockout plants. All knockout plants contained two lodicules and supernumerary carpels. (A) HvSL1-1, (B) HvSL1-3, (C) HvSL1-5, (D) HvSL1-6, and (E) HvSL1-10. Partial stamen conversions (sc) are annotated. Scale bars: 100 µm.

To provide additional support for *HvSL1* being the causative gene underlying the *mov2.g* phenotype, *Hvsl1* knockout plants were generated by CRISPR/Cas9 editing. Transformation of immature barley embryos (cv. Golden Promise) led to the generation of five T_0_ lines—HvSL1-1, HvSL1-3, HvSL1-5, HvSL1-6, and HvSL1-10—that phenocopied *mov2.g* plants. Specifically, HvSL1-6 was found to be a homo-allelic edit while HvSL1-1, HvSL1-3, and HvSL1-5 were all bi-allelic and HvSL1-10 was hetero-allelic (Supplementary Fig. S7). In all cases, edits occurred at the canonical cut site and resulted in loss-of-function frameshift mutations. The resulting phenotypes differed among lines, reflecting the phenotypic variation also observed in *mov2.g* plants. While all retained correct lodicule development, there was variation in the degree of stamen to carpel conversion, as well as in the number and complexity of supernumerary carpel-like structures (Fig. 4).

### HvSL1 influences *HvMADS16* expression but not that of other B-class genes

Since the early 1990s, flower development has always been described in the framework of the ABC model, whereby specific classes of genes are needed for the correct specification of each floral organ (Coen and Meyerowitz, 1991). The abnormal development of stamens and carpels in *mov2.g* is consistent with altered activity of B-class genes. Using a homology-based search, three B-class genes were found in barley, consisting of two GLO-like homologues (referred to here as *HvMADS2* and *HvMADS4*) and a single DEF-like homologue (referred to as *HvMADS16*), confirming already published data (Callens *et al*., 2018). These genes were evaluated in further detail.

B-class proteins can act in either homodimeric or heterodimeric complexes involving DEF and GLO proteins (Winter *et al*., 2002). To assess interactions among the barley B-class proteins, BiFC was conducted in onion epidermal cells. Fluorescence signal was observed only in cells co-expressing *HvMADS16* with *HvMADS2* or *HvMADS4* (Supplementary Fig. S8). For these combinations, fluorescence was predominantly confined to the nucleus. No signal was observed when the two GLO-like genes *HvMADS2* and *HvMADS4* were co-expressed in the same cells, or with a truncated version of *HvMADS16* (*ΔHvMADS16-nYFP*), containing only the MADS-domain. Likewise, no signal could be detected when homodimeric combinations for each B-class gene were tested (Supplementary Fig. S8).

**Fig. 5.**
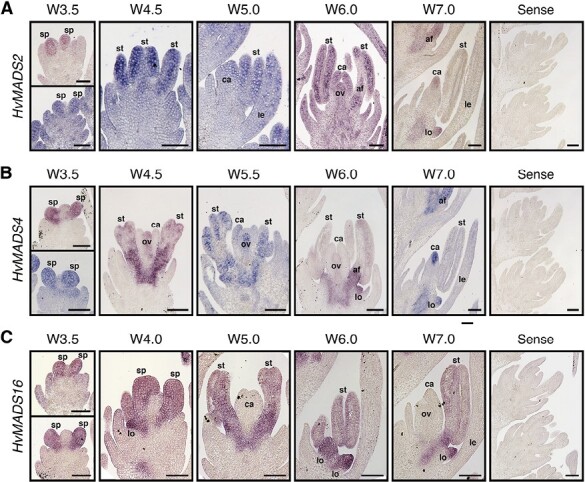
Expression pattern as assessed by *in situ* hybridization for barley B-class genes. (A) *HvMADS2*, (B) *HvMADS4*, and (C) *HvMADS16*. Annotations indicate stamen primordia (sp), lemma (le), lodicule (lo), stamen (st), anther filament (af), carpel (ca), and ovule (ov). Scale bars: 100 µm.

To determine whether heterodimerization is consistent with co-located gene expression, the expression patterns of the B-class genes were assessed by *in situ* hybridization in developing inflorescences and florets (Fig. 5). In the wild type (cv. Steptoe), *HvMADS2* was found to be expressed in most floral tissues (glumes, lodicules, stamen primordia, and carpel) in the early stages (W3.5–W6.0), but the signal became more specific as development progressed (Fig. 5A). In stamens, expression was initially localized mainly in the locules, but was later restricted to the filament (W7.0) (Fig. 5B). *HvMADS4* showed a similar pattern; however, it was also observed to accumulate in the ovule primordium (W4.5–W6.0). At maturity, both *HvMADS2* and *HvMADS4* were detected at the apex of the carpel, as well as in the lodicules. Expression of *HvMADS16* was first detected in the stamen and lodicule primordia (W3.5) (Fig. 5C). As the stamens develop, *HvMADS16* signal became weaker, while remaining strong in the lodicules (W6.0–W7.0). No expression was observed in the carpel or ovule at any stage and no signal was detected with the sense probes.

The overlapping expression domain of *HvSL1* and B-class genes, together with the specific *mov2.g* phenotype, suggests that these genes may act in a common pathway. Moreover, studies conducted in rice suggest that OsSL1 acts as an upstream positive regulator of *OsMADS16* transcription (Xiao *et al*., 2009). Expression assays in isolated barley protoplasts were performed to investigate a potential interaction between HvSL1 and barley B-class genes (Supplementary Fig. S9). Independent transfection experiments showed that protoplasts carrying a *p35S:HvSL1* construct showed higher *HvSL1* expression relative to protoplasts transfected with a mock solution (Supplementary Fig. S9B). In addition, a qRT-PCR assay indicated that endogenous *HvMADS16* transcript abundance was significantly increased in transfected protoplasts compared with the mock, while *HvMADS2* and *HvMADS4* expression remained unchanged (Supplementary Fig. S9C). This suggests that HvSL1 may also act as a positive regulator of *HvMADS16* in barley.

### The B-class gene *HvMADS16* is absent in *mov1* plants

**Fig. 6.**
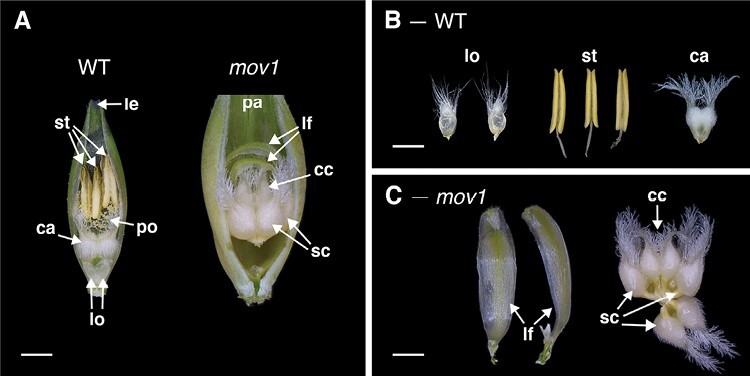
Florets and floral organs in the wild type (WT) and *mov1*. (A) Exposed WT and *mov1* florets. Palea or lemma have been removed to show internal floral organs. (B) The floral organs in a WT floret consist of two lodicules, three stamens, and one carpel. (C) In *mov1* florets, the lodicules are converted into bract-like organs, and stamens are converted into additional carpels. Scale bars: 1000 µm. Lemma (le), palea (pa), lodicule (lo), stamen (st), carpel (ca), supernumerary carpel-like structure (sc), central carpel (cc), leaf-like organ (lf), pollen (po).

To further dissect the relationship between HvSL1 and *HvMADS16*, we aimed to create a *Hvmads16* mutant using CRISPR/Cas9. Despite multiple attempts with different guides, edited plants were not recovered. To overcome this, we again investigated historical induced mutant resources. At least three multiovary loci have been reported to date (Franckowiak and Lundqvist, 2013). Of these, the *mov1* locus (Tazhin, 1980; Soule *et al*., 2000) bears a striking resemblance to the rice *Osmads16/superwoman1* mutant (Nagasawa *et al*., 2003). Under our growth conditions, *mov1* florets consistently showed direct homeotic transformation of lodicules into leaf-like organs and of stamens into carpels (Fig. 6). In each instance, the palea, lemma, and central carpel were retained. Unlike *mov2.g/Hvsl1* mutants, the four carpel-like organs were sterile as the female gametophyte failed to differentiate correctly (Supplementary Fig. S10). Furthermore, *mov1* florets did not produce any grains when manually cross-pollinated with wild-type pollen.

**Fig. 7.**
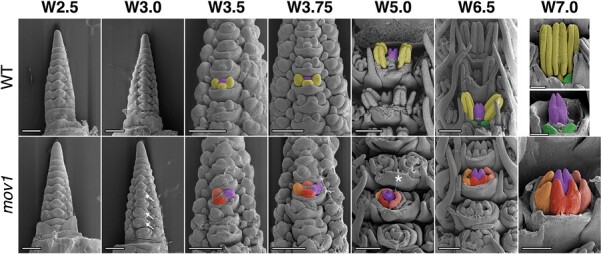
SEM of *mov1* inflorescence development. In the wild type (WT), primordia giving rise to stamens are false-coloured in yellow, cells giving rise to the carpel in purple, and lodicules in green. In each panel from W3.5 onwards, the primordia are false-coloured in a single floret to enable comparison between genotypes. In *mov1*, the central carpel (purple) is retained while the stamens are converted into additional carpels. Cells giving rise to the additional carpels are false-coloured in different shades of red/orange. White arrows in *mov1* (W3.0) indicate creases in the floral meristems. The white star at W5.0 indicates separation of a single floral meristem into two distinct florets; for greater detail, see Supplementary Fig. S11. Waddington stage is indicated for each developmental time point; from W5.0, lemma and/or stamens have been removed to expose the carpels. Scale bars: 200 µm.

SEM images of wild-type (cv. Steptoe) and *mov1* developing inflorescences were compared to determine the effect of *mov1* on floral organ development (Fig. 7). The earliest observable difference was seen immediately preceding the appearance of stamen primordia at W3.0, whereby a crease appeared in the basal floral meristems of *mov1* inflorescences. At stage W3.5–W3.75, instead of developing dome-shaped stamen primordia, the meristems in *mov1* divided into irregularly shaped protrusions which arranged into discernible multiple concentric creases by stage W5.0. As development progressed (W6.5–W7.0), each crease developed into a carpel, eventually leading to the four-carpel structure visible in the mature *mov1* floret. Occasionally, it was observed that a single floral meristem could give rise to two distinct florets (Fig. 7; Supplementray Fig. S11).

To determine whether mutation of a B-class gene contributes to the *mov1* phenotype, a PCR-based strategy, consisting of PCR amplification followed by Sanger sequencing of the amplicon, was used to survey all B-class genes for presence/absence, and structural and sequence variants in *mov1* plants relative to the wild type (cv. Steptoe). *HvMADS2* and *HvMADS4* did not show differences between genotypes when tested for amplicon size by PCR or polymorphisms by sequencing. In contrast, *HvMADS16* was not detected in *mov1* mutants (Supplementary Fig. S12A). Genotyping by copy number analysis combined with phenotyping demonstrated that absence of *HvMADS16* co-segregated perfectly with the *mov1* phenotype and that *mov1* segregates as a single recessive Mendelian locus (3:1, wild type:multiovary) (Supplementary Table S14). These findings indicate that *mov1* lacks *HvMADS16*.

To define the size of the deletion surrounding *HvMADS16*, a PCR-based approach was used to test the presence/absence of neighbouring gene sequences based on annotations from the barley reference Morex genome Hv_IBSC_PGSB_v2. Overall, the *mov1* mutant appeared to be missing a region of ~0.95 Mb relative to the wild type (Supplementary Fig. S12C). According to the reference sequence, this region is predicted to include three gene sequences: *HORVU7Hr1G091190* (40S ribosomal protein), *HORVU7Hr1G091200* (undescribed protein); and *HvMADS16* (Supplementary Table S15). Consistent with a role for *HvMADS16* in inflorescence specification, transcripts were predominantly detected in developing wild-type inflorescences when tested by qRT-PCR across a cv. Steptoe tissue series (Supplementary Fig. S12B). On the other hand, transcripts for *HORVU7Hr1G091200* could not be detected in any of the tissues examined. Furthermore, a BLASTx query against NCBI non-redundant protein databases found no significant similarity in other species, suggesting tht *HORVU7Hr1G091200* is a likely pseudogene. The presence of *HORVU7Hr1G091190* encoding a 40S ribosomal protein could not be uniquely assayed due to the highly repetitive nature of its sequence. However, publicly available RNA-seq data (Colmsee *et al*., 2015) indicate that *HORVU7Hr1G091190* is not expressed in a range of barley tissues including the inflorescence. Considering the specific homeotic conversion of floral organs in whorls 2 and 3, the absence of *HvMADS16* in *mov1* mutant plants and the role of B-class genes in other plant species, *HvMADS16* appears to be the most likely causal agent for *mov1*.

### *HvSL1* and *HvMADS16* differentially affect expression of floral regulators

**Fig. 8.**
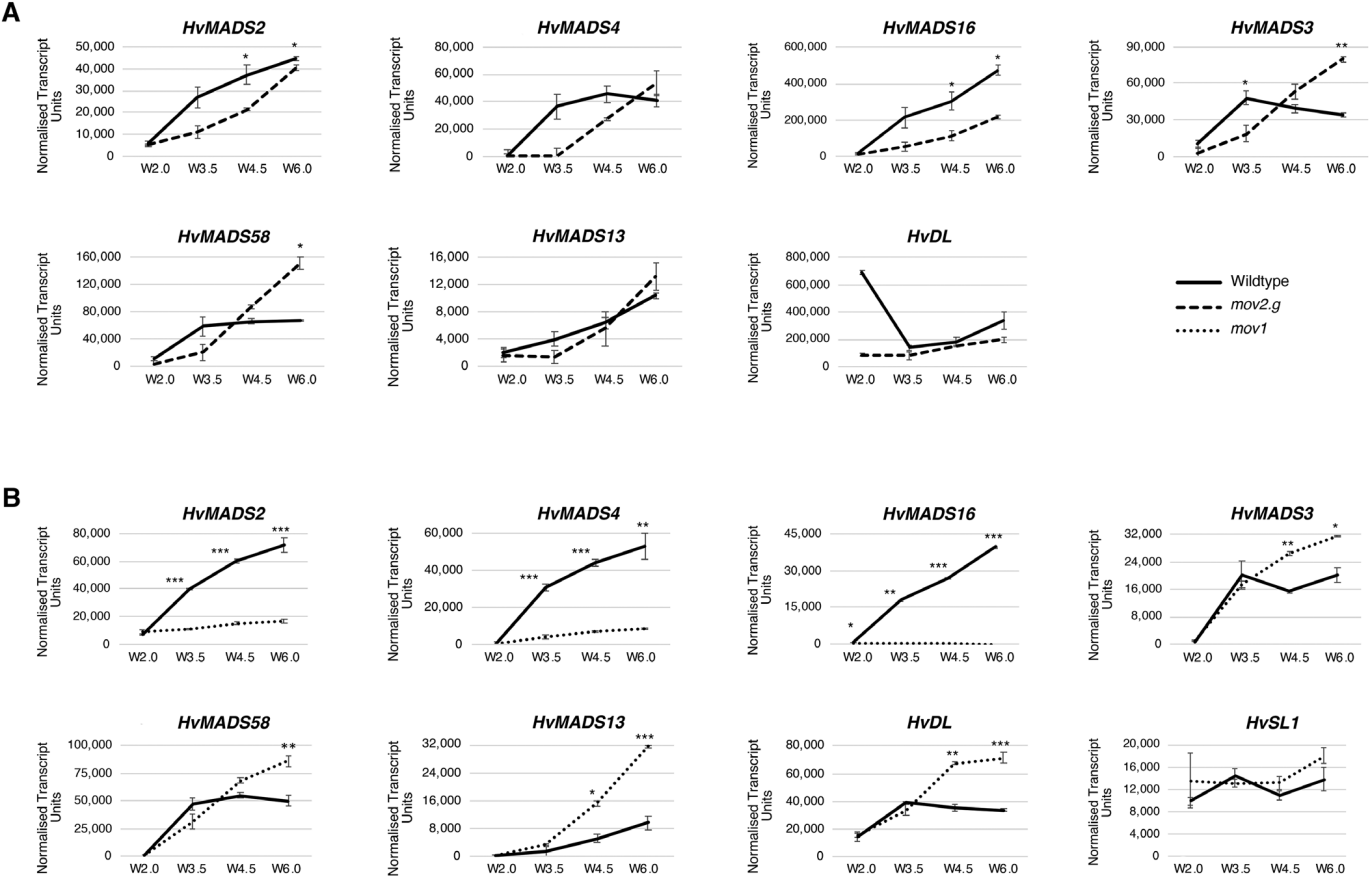
Transcript abundance of floral genes assessed by qRT-PCR in *mov2.g* and *mov1* developing inflorescences. Gene expression of B-class genes (*HvMADS2*, *HvMADS4*, *HvMADS16*), C-class genes (*HvMADS3*, *HvMADS58*), D-class gene *HvMADS13*, and carpel genes *HvDL* and *HvSL1* was assayed in (A) *mov2.g* and (B) *mov1* inflorescences at stages W2.0 (double ridge), W3.5 (stamen primordia), W4.5 (carpel primordium), and W6.0 (stamen and carpel development). Error bars represent ±SE. For each time point, two-tailed *t*-test *P*-values ≤0.05 (*), ≤0.005 (**), and ≤0.001 (***) are shown for differences between the wild type and *mov1*. For each sample, *n*=3 independent biological replicates.

To establish how the *HvSL1* (*mov2.g*) and *HvMADS16* (*mov1*) deletions might influence molecular pathways underlying stamen and carpel formation, qRT-PCR was performed on developing inflorescences from the wild type, *mov2.g*, and *mov1* at stages W2.0–W6.0 (Fig. 8), which encompass the specification and differentiation of the reproductive organs.

When comparing transcript abundance for the three B-class genes in *mov2.g* (Fig. 8A), the most pronounced difference was observed for *HvMADS16*, which was significantly reduced in the mutant. However, despite an overall reduction of transcript abundance in *mov2.g*, expression still increased with development. A similar trend was observed for *HvMADS2*, while for *HvMADS4*, transcript abundance remained lower in *mov2.g* only until stamen primordia specification (stage W3.5). After stage W3.5, transcript abundance for *HvMADS4* steadily increased to match wild-type levels by stage W6.0. For C-class *HvMADS3* and *HvMADS58* MADS-box genes, transcript abundance in *mov2.g* showed a delayed accumulation relative to the wild type, followed by an increase eventually exceeding wild-type levels. Conversely, transcript abundance of the ovule-specific D-class gene *HvMADS13* and the barley orthologue of the rice carpel-specific YABBY transcription factor *HvDL* (*DROOPING LEAF*) remained largely unaffected in *mov2.g* samples.

**Fig. 9.**
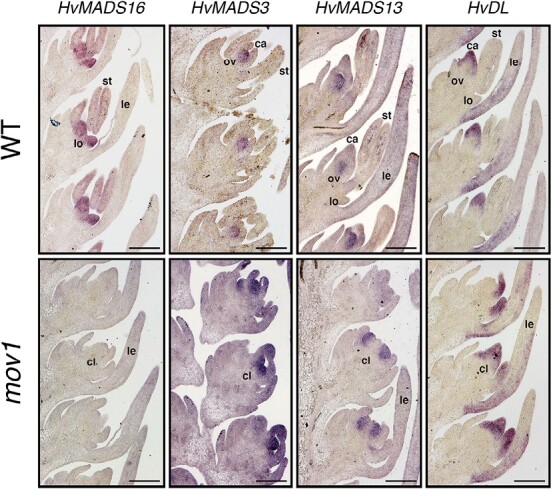
Spatial expression of floral homeotic genes in wild-type (WT) and *mov1* inflorescences. Expression patterns as detected by *in situ* hybridization are shown in WT and *mov1* inflorescences at stage W6.0 for genes *HvMADS16*, *HvMADS3*, *HvMADS13*, and *HvDL*. Lemma (le), lodicule (lo), stamen (st), carpel (ca), ovule (ov), and carpel-like structure (cl). Scale bars: 200 µm.

In *mov1* (Fig. 8B), *HvSL1* expression did not significantly differ from that in the wild type. In contrast, expression of the B-class genes *HvMADS2* and *HvMADS4* was significantly lower in *mov1* from stage W3.5 onwards, and *HvMADS16* expression was absent. Conversely, expression of the C-class genes (*HvMADS3* and *HvMADS58*), a D-class gene (*HvMADS13*), and the carpel-specific *HvDL* gene was significantly increased in *mov1* compared with the wild type following stamen primordia specification (W3.5). To determine the spatio-temporal expression pattern during floret development, *in situ* hybridization of selected genes was performed in wild-type (cv. Steptoe) and *mov1* mature inflorescences (Fig. 9). As expected, *HvMADS16* expression was undetectable in *mov1* inflorescences in any floral organ, whereas it was expressed in wild-type lodicules and stamens. The expression patterns of *HvMADS3* (C-class) and *HvMADS13* (D-class) genes were found to be very similar: in wild-type florets, expression was confined to the ovule. In *mov1*, both *HvMADS3* and *HvMADS13* were also expressed in the primordia of ectopic ovules. Notably, *HvDL* expression in the wild type was restricted to the carpels and to the abaxial side of the lemma but was absent in lodicules or stamens. In *mov1* florets, *HvDL* expression remained in the lemma and was present in the central and ectopic carpels. For all genes tested, sense probes gave no observable signal (Supplementary Fig. S13).

## Discussion

### Deletion of the zinc-finger transcription factor HvSL1 underlies the *mov2.g* multiovary phenotype

The present study demonstrates that *HORVU3Hr1G003740* on chromosome 3H underlies the barley *mov2.g* locus. *HORVU3Hr1G003740*, referred to here as *HvSL1*, encodes a putative C2H2 zinc-finger transcription factor and is the likely orthologue of rice *STAMENLESS1* (*OsSL1*) (Xiao *et al*., 2009). Indeed, the *mov2.g* multiovary phenotype closely resembles that of *sl1* mutants in rice. Both barley and rice mutants exhibit a varied number of irregularly shaped carpels at the expense of stamens, and ectopic organs are present predominantly in whorl 3, while whorl 4 remains less affected and typically retains a single carpel (Xiao *et al*., 2009). Despite this, notable phenotypic differences are present between *mov2.g* and *sl1*. Unlike rice *sl1*, a high rate of abnormal or converted lodicules was not observed in *mov2.g* plants. Likewise, palea/lemma-shaped organs or undifferentiated tissue were not observed among the ectopic carpels in whorl 3 of *Hvsl1*. In addition, inflorescence meristem fasciation appears to be a unique feature of *mov2.g*, although this requires confirmation by further study.

Moreover, the most prominent feature of *mov2.g* spikes is the ability to produce multiple grains per floret upon cross-pollination (Fig. 1D, E). This is in accord with initial reports of barley multiovary *mo* mutants (Moh and Nilan, 1953; Kamra and Nilan, 1959), proposed to be allelic to *mov2.g* (Franckowiak and Lundqvist, 2013). However, production of multiple grains per floret has not been reported for rice *sl1*.

More pronounced functional differences are present between *HvSL1* and the respective Arabidopsis orthologues, *JAG* and *NUB*. Both *JAG* and *NUB* appear to be involved in correct stamen and carpel development; however, lack of function in these genes does not alter organ identity, but rather organ morphogenesis (Dinneny *et al*., 2006). Furthermore, the role of *JAG* and *NUB* as shape determinants is not only restricted to the reproductive organs but also affects the outer floral whorls as well as vegetative tissues, indicating a more general role in lateral organ shape (Ohno *et al*., 2004). This broad comparison indicates that the function of barley *HvSL1* is restricted to controlling specific floral organ identity in the *Triticeae* relative to both closely related monocots (rice) and the more distant dicots (Arabidopsis). Given that *mov2.g* plants only show conversion of whorl 3 organs (stamens) into supernumerary carpels (Fig. 1), we propose that *HvSL1* is necessary for correct stamen development.

### Absence of *HvMADS16* underlies the *mov1* multiovary mutation

The current study positioned the *mov1* locus at higher resolution on chromosome 7H and significantly narrowed the critical interval to only three putative genes. Among these, the MADS-box transcription factor gene *HvMADS16* emerges as the most likely candidate for *mov1*. Indeed, the *mov1* phenotype is remarkably similar to characterized B-class mutants such as Arabidopsis *apetala3* (*ap3*) (Jack *et al*., 1992), rice *superwoman1* (*spw1*) (Nagasawa *et al*., 2003), and maize *silky1-5* (*si1-5*) (Ambrose *et al*., 2000) which all show transformation of petals/lodicules and stamens into bract-like and carpel-like organs, respectively, and involve DEF-like genes. Additional molecular evidence supports our hypothesis that altered *HvMADS16* function underlies the *mov1* phenotype. Besides being physically absent in *mov1* (Supplementary Fig. S12A), *HvMADS16* is preferentially expressed in inflorescences (Supplementary Fig. S12B). Within wild-type developing florets, *HvMADS16* expression is specifically confined to lodicules and stamens (Fig. 5C), similarly to *SPW1* expression in rice (Nagasawa *et al*., 2003). The specific expression pattern is also consistent with the observation that *mov1* only shows a major phenotype in floral development, but not in any other aspect of plant growth.

Another interesting *mov1* phenotype is complete female sterility, as was observed for the orthologous rice mutant *spw1* (Nagasawa *et al*., 2003). Histological analysis suggested that the ovules in all four *mov1* carpels are unable to produce a fully differentiated female gametophyte (Supplementary Fig. S10). Instead, the ovule-like structures are filled with undifferentiated cell layers. This phenotype is distinct from *mov2.g*, which often produced multiple fertile ovules, but is similar to that observed in wheat pistillody lines (Yamada *et al*., 2009) and in rice *spw1* (Nagasawa *et al*., 2003), suggestive of a potentially conserved role for HvMADS16 in ovule development. Interestingly, this contrasts with the Arabidopsis orthologue *ap3* mutant that produces viable seeds (Jack *et al*., 1992), and with maize *si1-5* in which only the transformed carpels in the tassels (the male florets) were reported to be sterile (Ambrose *et al*., 2000). It also contrasts with the lack of *HvMADS16* expression in ovules, and is suggestive of an indirect non-cell-autonomous effect, which might be considered in future studies.

### *HvSL1* and *HvMADS16* functionally overlap during floral specification

As revealed by SEM analysis, phenotypic differences in *mov2* and *mov1* inflorescences appear very early in floret development (Figs 2 and 7). This is consistent with *HvSL1* and *HvMADS16* being expressed from double ridge (W2.0) and gradually increasing expression until differentiation of the reproductive organs (W6.0) in wild-type inflorescences (Supplementary Figs S6B, 8).

Studies in rice have suggested that OsSL1 may act as a positive upstream regulator of *OsMADS16* (Xiao *et al*., 2009). Considering that *HvSL1* expression temporally precedes that of *HvMADS16* and that their overlapping expression domains overlap, we hypothesized that *HvSL1* might regulate B-class genes in barley, particularly *HvMADS16*. Indeed, the presence of HvSL1 appears to positively influence endogenous *HvMADS16* expression in transfected protoplasts (Supplementary Fig. S9C). Moreover, expression of *HvMADS16* is down-regulated in *mov2.g* even before stamen primordia initiation, whereas the expression profile of *HvSL1* does not significantly differ from that of the wild type in *mov1* plants (Fig. 8). These results suggest a conserved regulatory network with rice in controlling stamen identity. Further study is required to establish whether HvSL1 directly regulates *HvMADS16* or acts through an intermediate.

### Absence of *HvSL1* and *HvMADS16* disrupts the expression of B-class genes

The absence of *HvMADS16* and *HvSL1* in *mov1* and *mov2.g* influenced the expression of the transcription factors driving floral development. As expected, there is no expression of *HvMADS16* in *mov1* (Fig. 8B). Expression of the other B-class genes (*HvMADS2* and *HvMADS4*) in *mov1* is significantly reduced from a very early stage onwards, suggesting a tight regulatory relationship between these three genes (Fig. 8B). Evidence supporting this hypothesis comes from the overlapping expression of B-class genes in barley lodicules and stamens (Fig. 5), and the obligate interaction of HvMADS16 (DEF-like) with either GLO-like protein (HvMADS2 and HvMADS4) (Supplementary Fig. S8). These findings are consistent with reports in other species whereby obligate DEF–GLO heterodimers can activate their own expression and initiate a positive autoregulatory feedback loop (Schwarz-Sommer *et al*., 1992; Goto and Meyerowitz, 1994; McGonigle *et al*., 1996; Bartlett *et al*., 2015).

The reduced expression of all three B-class genes *HvMADS2*, *HvMADS4*, and *HvMADS16* in *mov2.g* inflorescences is consistent with the lack of stamens (Fig. 8A). Nevertheless, expression of these genes is not completely abolished and is sufficient to drive normal lodicule development in whorl 2. Indeed, transcript abundance of both *HvMADS2* and *HvMADS4* reaches wild-type levels by stage W6.0. The low levels of *HvMADS2* and *HvMADS4* at earlier stages could indicate a slower initiation of the positive autoregulatory feedback loop typical of B-class genes, due to the low *HvMADS16* levels.

### *HvSL1* and *HvMADS16* have distinct effects on C- and D-class genes

The expression of C-class genes (*HvMADS3* and *HvMADS58*), D-class genes (*HvMADS13*), and *HvDL* increased in *mov1* inflorescences (Fig. 8B). In wild-type barley, these genes are expressed in the fourth whorl and are predicted to specify carpel (*HvMADS58* and *HvDL*) and ovule (*HvMADS3* and *HvMADS13*) development. The *HvDL* expression pattern is consistent with that of its rice orthologue *OsDL* (Nagasawa *et al*., 2003), and partly with that of the maize co-orthologues *drl1* and *drl2* (Strable and Vollbrecht, 2019). Whereas transcript of maize *drl* genes accumulated in the carpel primordia and lemma as well as in the palea, glumes, and cryptic bracts (Strable and Vollbrecht, 2019), expression of rice *OsDL* was shown to be confined to the carpel and to the central vein of the lemma (Ohmori *et al*., 2011). Similarly, *HvDL* expression in both the wild type and *mov1* was also restricted to the central carpel, ectopic carpels, and the base of the lemma (Fig. 9), overall suggesting a conserved function in carpel morphogenesis among grasses. Likewise, *HvMADS13* signal in wild-type barley ovules is similar to that of *OsMADS13* in rice (Lopez-Dee *et al*., 1999), whereas the specific expression pattern of *HvMADS3* in ovules highlights a potential subfunctionalization of C-class genes in the grasses. Both the rice (*OsMADS3*) and maize (*ZMM2*) *HvMADS3* orthologues have been reported to be strongly expressed in stamen primordia (Dreni *et al*., 2011) and anthers (Mena *et al*., 1996), respectively. *OsMADS3* plays an important role in specifying stamen identity, as well as in repressing lodicule formation (Yamaguchi *et al*., 2006). The ovule-specific *HvMADS3* expression (Fig. 9), and the increased transcript abundance detected in *mov1* (Fig. 8B) argue against a conserved role for *HvMADS3* in stamen specification in barley.

In *mov1* inflorescences, the increased transcript levels of both *HvMADS3* and *HvMADS13* can be explained by the additional ovule structures observed by histochemical analysis (Supplementary Fig. S10) and corresponds well to the spatial expression of both genes in the mutant (Fig. 9). However, expression of these ovule identity genes is apparently insufficient to drive correct ovule formation in *mov1*, suggesting that their activity may be compromised, or that ovule fertility defects are manifested later during gametogenesis. We speculate that the increased transcript levels of DL-, C-, and D-class genes in *mov1* can be explained by a reduced repressive action of B-class genes on carpel- and ovule-promoting genes, together with the expansion of these genes to the third whorl, resulting in florets with carpels in two consecutive whorls (whorl 3 and whorl 4).

In *mov2.g*, the increased abundance of carpel- and ovule-specific C-class genes (*HvMADS3* and *HvMADS58*) (Fig. 8A) is also consistent with the presence of supernumerary carpels (Fig. 1). Despite this, expression levels of the D-class gene *HvMADS13* and the carpel-specific *HvDL* gene remain unaffected in *mov2.g* with respect to the wild type (Fig. 8A). This contrasts with *mov1* and confirms that an increase in ovule number alone does not always relate to increased *HvMADS13* and *HvDL* expression. A possible explanation is that these two genes may play a minor role in the formation of additional ovules and carpels in *mov2.g* compared with other genes. Alternatively, HvDL in *mov2.g* may have slower expression dynamics. Indeed, most of the investigated genes seem to have a delayed response in *mov2.g* compared with the wild type.

### A model for barley stamen specification

Although the ABC model was initially proposed as a universal model to explain flower development, differences have been found across plant species, even among members of the same family, as is the case between rice and barley (*Poaceae*). For example, some MADS-box genes have acquired grass-specific functions as seen for the A-class genes in rice which specify palea and lodicules (reviewed in Callens *et al*., 2018). Other classes underwent partial subfunctionalization, as is the case of the C-class genes in rice (Kang *et al*., 1995; Yamaguchi *et al*., 2006; Kuijer *et al*., 2021) and maize (Ali *et al*., 2019), or the D-lineage in *Brachypodium distachyon* (Wei *et al*., 2013).

In barley, an expression atlas throughout inflorescence development of 34 MIKc MADS genes highlighted how some transcription patterns strongly deviate from the predicted model (Kuijer *et al.*, 2021). Indeed, several barley MADS-box genes have acquired roles in addition to floral specification. This is the case for *HvMADS14* (A-class) which controls vernalization-induced flowering, acting as the *VERNALIZATION1* equivalent in other temperate cereals (Trevaskis *et al*., 2003). Another example is *HvMADS1* (E-class), which has been recently reported to direct plant thermomorphogenesis (Li *et al*., 2021). Conversely, the core B-class function, particularly stamen development, appears to be extremely conserved among domesticated grasses.

**Fig. 10.**
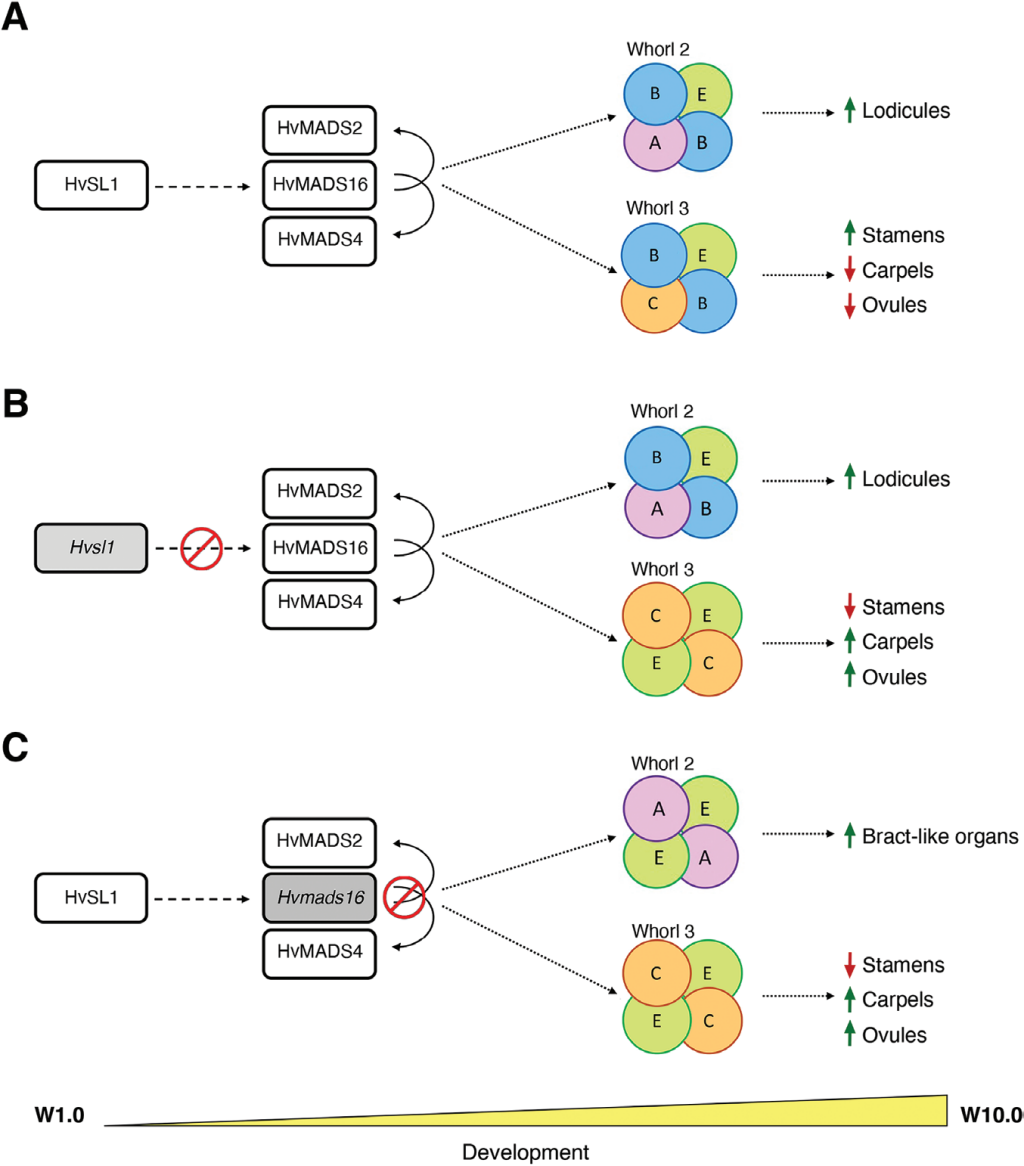
Model for barley stamen specification. (A) As inflorescence development progresses in the wild type, genes of the ABC model combine in floral quartets to specify lodicules in whorl 2 and stamens in whorl 3, with putative repression of carpel and ovule-specific genes. The absence of (B) *HvSL1* in *mov2.g* plants and (C) of *HvMADS16* in *mov1* plants affects the balance and composition of the floral quartets that form in these whorls, leading to an altered specification of the resulting floral organs. Within the floral quartets, B-class genes act as obligate GLO (HvMADS2 and HvMADS4)/DEF (HvMADS16) heterodimers. Dashed arrows indicate direct or indirect interaction, solid arrows indicate direct interactions found in this study, while dotted arrows indicate a process or consequence. For each mutant, the missing protein is indicated in grey, and the red symbol indicates the affected interaction.

Based on the results obtained in this study, together with previous reports in barley and other plant species, we formulate a testable model for barley stamen specification (Fig. 10). In this model, HvSL1 acts predominantly in whorl 3 and promotes *HvMADS16* in the wild type (Fig. 10A). The B-class genes *HvMADS16*, *HvMADS2*, and *HvMADS4* are expressed in floral whorls 2 and 3 and form DEF–GLO heterodimers. Based on the floral quartet model, the heterodimers associate in higher order protein complexes with A- and E-class genes, leading to the formation of lodicules in whorl 2. In whorl 3, instead, the floral quartet complex forms between B-, C-, and E-class genes, promoting stamen development while concomitantly repressing carpel formation.

The lack of *HvSL1* in *mov2.g* plants results in a shift in this balance (Fig. 10B). At a molecular level, we speculate that B-class activity in *mov2.g* florets is predominantly maintained in whorl 2 where enough HvMADS16 is present to develop normal lodicules. At the same time, the reduced levels of HvMADS16 in whorl 3 are insufficient to successfully form functional B-class heterodimers. Consequently, the predominant quaternary complexes forming in whorl 3 are between C- and E-class genes, which promote carpel formation. Concomitant expansion of ovule-promoting genes such as *HvMADS3* to whorl 3 may then explain multiple carpels having a functional female gametophyte.

In *mov1*, *HvMADS16* is absent. The remaining B-class GLO-like proteins HvMADS2 and HvMADS4 do not interact and therefore cannot compensate for the absence of HvMADS16. Thus, the only protein complexes that can form in whorl 2 are between A- and E-class genes, and between C- and E-class genes in whorl 3. As a result, bract-like organs develop in whorl 2 and carpel formation is promoted in whorl 3, leading to the multiovary phenotype observed in *mov1* (Fig. 10C).

In conclusion, we have characterized two barley multiovary loci, *mov2* and *mov1*, and identified likely causative genes, namely a C2H2 zinc-finger transcription factor gene termed here *HvSL1*, and the B-class gene *HvMADS16*. Although both mutants exhibit a similar multiovary phenotype, the difference in fertility is remarkable: while *mov1* is completely sterile, *mov2* can produce multiple grains per floret. This indicates that the presence of multiovary is not necessarily indicative of fully functional reproductive development in barley. Studying the molecular changes underlying these differences will improve our understanding of floral development at a species-specific level and expand the tools available for modification of cereal florets for breeding.

## Supplementary data

The following supplementary data are available at *JXB* online.

Table S1. Primer sequences and Taqman probes used for copy number analysis to genotype *mov2.g* and *mov1* plants.

Table S2. Sequence of KASP™ markers on chromosome 3H used to map the *mov2* locus.

Table S3. PCR primer sequences for testing the presence of genes upstream and downstream from *HvSL1* on chromosome 3H.

Table S4. PCR primer sequences for testing the presence of barley B-class genes.

Table S5. PCR primer sequences for testing the presence of genes upstream and downstream from *HvMADS16* on chromosome 7H.

Table S6. qRT-PCR primer sequences.

Table S7. Primer sequences for *HvSL1* CRISPR knockout.

Table S8. Primer sequence for cloning of *in situ* hybridization antisense and sense probes.

Table S9. Primer sequences for BiFC cloning.

Table S10. Floral organ frequency in the wild type and *mov2.g*.

Table S11. Annotated genes present in the mapped *mov2* critical interval between flanking markers chr3H_9748112 and chr3H_10289104.

Table S12. BLASTp results using barley HvSL1 as query against the rice genome.

Table S13. BLASTp results using rice OsSL1 as query against the barley genome.

Table S14. Observed segregation ratios of *mov1* phenotype in heterozygous growing material.

Table S15. Genes on chromosome 7H tested by PCR.

Fig. S1. Histological sections of *mov2.g* carpels.

Fig. S2. Spike morphology in wild-type and *mov2.g* plants.

Fig. S3. Mapping of the *mov2* locus in a *mov2.g*×Morex bi-parental population.

Fig. S4. Heatmap of gene expression for genes in the critical *mov2* interval.

Fig. S5. *HvSL1* and *OsSL1* gene models and alignment.

Fig. S6. *HvSL1* deletion in *mov2.g*.

Fig. S7. CRISPR design and analysis of *Hvsl1* knockout plants.

Fig. S8. BiFC assays showing interaction between barley B-class genes.

Fig. S9. Transfection efficiency and transcript abundance in barley protoplasts.

Fig. S10. Histological sections of *mov1* carpels.

Fig. S11. Details of *mov1* inflorescence development.

Fig. S12. Characterization of the *mov1* deletion and *HvMADS16*.

Fig. S13. *In situ* hybridization with sense probes on wild-type and *mov1* inflorescences.

erad218_suppl_supplementary_Tables_S1-S5_figures_S1-S13

## Data Availability

All data supporting the findings of this study can be found within the paper and its supplementary data published online. They are also available from the corresponding author, Matthew R. Tucker, upon request.
