# Supplementary material for: HvSL1 and HvMADS16 promote stamen identity to restrict multiple ovary formation in barley
Source: J Exp Bot. 2023 Jun 3;74(17):5039–56. doi: 10.1093/jxb/erad218 (PMC10498024; doi:10.1093/jxb/erad218)
Supplement: erad218_suppl_supplementary_Tables_S1-S5_figures_S1-S13 [file erad218_suppl_supplementary_tables_s1-s5_figures_s1-s13.pdf]

Supplementary Information

Title: *HvSL1* and *HvMADS16* promote stamen identity to restrict multiple ovary formation in barley

Authors: C. Selva, X. Yang, N. J. Shirley, R. Whitford, U. Baumann, M. R. Tucker

Corresponding Author

Associate Professor Matthew R. Tucker

Email: matthew.tucker@adelaide.edu.au

Phone: +61-8-83139241

Supplementary Information Legend

Supplementary Table S1. Primer sequences and Taqman probes used for copy number analysis to genotype *mov2.g* and *mov1* plants.

Supplementary Table S2. Sequence of KASP^{TM} markers on chromosomes 3H used to map the *mov2* locus.

Supplementary Table S3. PCR primer sequences for testing the presence of genes upstream and downstream from *HvSL1* on chromosome 3H.

Supplementary Table S4. PCR primer sequences for testing the presence of barley B-class genes.

Supplementary Table S5. PCR primer sequences for testing the presence of genes upstream and downstream from *HvMADS16* on chromosome 7H.

Supplementary Table S6. qRT-PCR primer sequences.

Supplementary Table S7. Primer sequences for *HvSL1* CRISPR knockout.

Supplementary Table S8. Primer sequence for cloning of *in situ* hybridization antisense and sense probes.

Supplementary Table S9. Primer sequences for BiFC cloning.

Supplementary Table S10. Floral organ frequency in wild-type and *mov2.g*.

Supplementary Table S11. Annotated genes present in the mapped *mov2* critical interval between flanking markers chr3H_9748112 and chr3H_10289104.

Supplementary Table S12. BLASTp results using barley HvSL1 as query against the rice genome.

Supplementary Table S13. BLASTp results using rice OsSL1 as query against the barley genome.

Supplementary Table S14. Observed segregation ratios of *mov1* phenotype in heterozygous growing material.

Supplementary Table S15. Genes on chromosome 7H tested by PCR.

Supplementary Fig. S1. Histological sections of *mov2.g* carpels.

Supplementary Fig. S2. Spike morphology of wild-type and *mov2.g* plants.

Supplementary Fig. S3. Mapping of the *mov2* locus in a *mov2.g* x Morex bi-parental population.

Supplementary Fig. S4. Heatmap of gene expression for genes in the critical *mov2* interval.

Supplementary Fig. S5. *HvSL1* and *OsSL1* gene models and alignment.

Supplementary Fig. S6. *HvSL1* deletion in *mov2.g*.

Supplementary Fig. S7. CRISPR design and analysis of *Hvsl1*-knockout plants.

Supplementary Fig. S8. BiFC assays showing interaction between barley B-class genes.

Supplementary Fig. S9. Transfection efficiency and transcript abundance in barley protoplasts.

Supplementary Fig. S10. Histological sections of *mov1* carpels.

Supplementary Fig. S11. Details of *mov1* inflorescence development.

Supplementary Fig. S12. Characterization of *mov1* deletion and *HvMADS16*.

Supplementary Fig. S13. *In situ* hybridization with sense probes on wild-type and *mov1* inflorescences.

Supplementary Table S1. Primer sequences and Taqman probes used for copy number analysis to genotype *mov2.g* and *mov1* plants. Fluorophore at 5' and 3' quencher are indicated for each probe.

Gene name	Gene ID	Primer sequences	Taqman probe
HvCO-like	*HORVU6Hr1G072620*	TGCTAACCGTGTGGCATCAC<br>GGTACATAGTGCTGCTGCATCTG	[HEX]CATGAGCGTGTGCGTGTCTGCG[BHQ1]
HvSL1	*HORVU3Hr1G003740*	GGAGGAGGAGGATTCAGGGGAGG<br>GCGTCGTGCTGTAGAGGTAGTGG	[FAM]TCGGAGACCCAAGCCACCACCCATT[BHQ1]
HvMADS16	*HORVU7Hr1G091210*	GTTTACCTTGCCTTGTGTCG<br>ACGAACTGCTTTCTCAAACG	[FAM]AGTTCTCCATGCCACTGCTCAAACACCA[BHQ1]

Supplementary Table S2. Sequence of KASP^{TM} markers on chromosomes 3H used to map the *mov2* locus. Flanking markers are indicated in bold. Marker order is based on the genetic map.

Marker name	Forward primer Allele 1	Forward primer Allele 2	Reverse Common primer	SNP
chr3H_28805649 [1]	GAAGGTGACCAAGTTCATGCTC GACGGTTCCATTCTGCAC	GAAGGTCGGAGTCAACGGATT GCTCGACGGTTCCATTCTGCAA	TCTTGATGCTGACATGT ATATGTTCTTGAT	[T/A]
chr3H_1006543 [1,2]	GAAGGTGACCAAGTTCATGCTA AGCTTGATTTCCACATGACCAA TTTC	GAAGGTCGGAGTCAACGGATT CTAAGCTTGATTTCCACATGACC AATTTT	ACTCCTCCCATGGCCTG ACCTT	[G/A]
chr3H_1367441 [2]	GAAGGTGACCAAGTTCATGCTC TCCGGATTCTTCAAGAGCTCT	GAAGGTCGGAGTCAACGGATT CTCCGGATTCTTCAAGAGCTCC	GCGTCGGAGGTGGGG AGGTT	[T/C]
ch3H_3865263 [2]	GAAGGTGACCAAGTTCATGCTG AAACCATATACCATAGCAGCAG CAA	GAAGGTCGGAGTCAACGGATT AAACCATATACCATAGCAGCAG CAG	CAAATGCTTTACTCATA ACGGCGGCAT	[A/G]
chr3H_7767159 [3]	GAAGGTGACCAAGTTCATGCTC GATCGGCGAGGACGAGACA	GAAGGTCGGAGTCAACGGATT GATCGGCGAGGACGAGACG	CCCACCGTCGAGACTC CGATA	[A/G]
chr3H_7767871 [2,3]	GAAGGTGACCAAGTTCATGCTA ACGTTGATGCTCTTCACCAATG GT	GAAGGTCGGAGTCAACGGATT CGTTGATGCTCTTCACCAATGGC	TTCAGCCTGAGCTTGA ATGGGACTT	[A/G]
chr3H_8709612 [3]	GAAGGTGACCAAGTTCATGCTG TGTGATGGACCGGCCTCGT	GAAGGTCGGAGTCAACGGATT GTGATGGACCGGCCTCGC	CTCCCTCCCAATGCACA CCGAT	[A/G]
chr3H_8787424 [3]	GAAGGTGACCAAGTTCATGCTA TGTAAAGTTTCTAGATGATGCAA GGC	GAAGGTCGGAGTCAACGGATT GATGTAAAGTTTCTAGATGATG CAAGGA	CATTTTCCAAGTTTCTT GCCCACAGTTTT	[G/T]
chr3H_8744266 [3]	GAAGGTGACCAAGTTCATGCTG GTCGTAAACAAATTATCTACCTGC	GAAGGTCGGAGTCAACGGATT GTGGTCGTAAACAAATTATCTA CCTGT	GGAATTTCGACCCCTG ACCATGTTA	[C/T]
chr3H_8969648 [3]	GAAGGTGACCAAGTTCATGCTC CCGTCTCCGTCGCCGG	GAAGGTCGGAGTCAACGGATT CCCGTCTCCGTCGCCGC	CTCCAGATGTCGTCGTC GTCGAA	[C/G]
chr3H_9095799 [3]	GAAGGTGACCAAGTTCATGCTA CGCGCAGGTGATGCACCG	GAAGGTCGGAGTCAACGGATT GACGCGCAGGTGATGCACCA	GCCCTCCACGGCGTCC TCTT	[G/A]
chr3H_9356192 [4]	GAAGGTGACCAAGTTCATGCTG GTTGCATCGATCGACGCCG	GAAGGTCGGAGTCAACGGATT CGGTTGCATCGATCGACGCCA	GCCCTTTTCTCTCCGGG CATCAT	[C/T]

chr3H_9347808 [4]	GAAGGTGACCAAGTTCATGCTCTATAGAGAATGGAGTAGTCTATAC	GAAGGTCGGAGTCAACGGATTGCTCTATAGAGAATGGAGTAGTCTATAT	CACAACAGGTAGGATAGCTAGAAACTATA	[G/A]
**chr3H_9748112 [4]**	**GAAGGTGACCAAGTTCATGCTATCAAACGCCAATCAAGGTTACTTTAC**	**GAAGGTCGGAGTCAACGGATTATCAAACGCCAATCAAGGTTACTTTAG**	**CCCTGAAATGAATAACCTTTTTTTAGGGAA**	**[C/G]**
**chr3H_10289104 [4]**	**GAAGGTGACCAAGTTCATGCTGGCGGCGGGAGGCTCTG**	**GAAGGTCGGAGTCAACGGATTGGCGGCGGGAGGCTCTC**	**CCCCGTCAAAGCCTCCCAAGAA**	**[G/C]**
chr3H_10601795 [4]	GAAGGTGACCAAGTTCATGCTGTTTGGGGCTTACTATGTGCCA	GAAGGTCGGAGTCAACGGATTGTTTGGGGCTTACTATGTGCCG	CCACACACGTGAACACRTTCAGGAT	[A/G]
chr3H_10797294 [4]	GAAGGTGACCAAGTTCATGCTATGTTGGCGACGCTTCTCCTC	GAAGGTCGGAGTCAACGGATTGATGTTGGCGACGCTTCTCCTA	TTTGAGCAGTAGCKGTGCAGCCAT	[C/A]
chr3H_11039299 [3]	GAAGGTGACCAAGTTCATGCTCCAGGTCTTCGAGTGCCCC	GAAGGTCGGAGTCAACGGATTCCAGGTCTTCGAGTGCCCG	GCCCGCAGGACTTGCAGGTTTT	[C/G]
chr3H_11335959 [3]	GAAGGTGACCAAGTTCATGCTCCGGCGTACGAGTACCCG	GAAGGTCGGAGTCAACGGATTCCGGCGTACGAGTACCCC	GTTACCGGGGRGCTGCTGGTT	[G/C]
chr3H_11617707 [3]	GAAGGTGACCAAGTTCATGCTACTTGCTCCAATAATCACAGCTCTC	GAAGGTCGGAGTCAACGGATTCACTTGCTCCAATAATCACAGCTCTA	AGGGTATTTGAAGATCGTATGGATCTCAT	[G/T]
chr3H_11702941 [3]	GAAGGTGACCAAGTTCATGCTGACAGCCGTTTCGTCGCCACA	GAAGGTCGGAGTCAACGGATTCAGCCGTTTCGTCGCCACG	CCGGCTTGTCTACGTCGTATATGAT	[A/G]
chr3H_17951104 [2]	GAAGGTGACCAAGTTCATGCTCACTTGTCGTTCTTGTCCTCCTC	GAAGGTCGGAGTCAACGGATTCACTTGTCGTTCTTGTCCTCCTT	TTGTCAGCGGCGAGCGCACTAA	[G/A]
chr3H_28161638 [2]	GAAGGTGACCAAGTTCATGCTCGTCTTTCCGCCCTGAGTTTG	GAAGGTCGGAGTCAACGGATTCCGTCTTTCCGCCCTGAGTTTA	GAAGTCGCCAGCTGTTGAAGTTCTT	[C/T]
chr3H_28805649 [2]	GAAGGTGACCAAGTTCATGCTCGACGGTTCCATTCTGCAC	GAAGGTCGGAGTCAACGGATTGCTCGACGGTTCCATTCTGCAA	TCTTGATGCTGACATGTATATGTTCTTGAT	[G/T]

[1] markers used to confirm heterozygosity of F_1 plants

[2] markers used to confirm *mov2* position

[3] markers used for mapping with F_2 segregants

[4] markers used for mapping with F_3 recombinants

Supplementary Table S3. PCR primer sequences for testing the presence of genes upstream and downstream of *HvSL1* (bold) on chromosome 3H.

Gene ID	Forward primer	Reverse primer
HORVU3Hr1G003690	GGTTACTTTACCCTTTCGATGTTCC	ACGAAGTAGTGCGTCCCGAAG
HORVU3Hr1G003760	CTAGCTAGCGAGCGCGTTATACC	TGGGAGGTCGATCTCATCAGTGC
HORVU3Hr1G003740	**CCAAACCAACACTTTAAGACTGC**	**TCTTATGGGGAGTAAAAAGGACC**
HORVU3Hr1G003810	GGGTGTAATCTGGTTGCTAATCC	ATCGACGTATCCTGATTCATTCC
HORVU3Hr1G003820	GTTTAGCAGTACGCATGAGACCC	AGCTAGTAGGGAGTCCTTGGAGG

Supplementary Table S4. PCR primer sequences for testing the presence of barley B-class genes.

Gene name	Gene ID	Forward primer	Reverse primer
HvGAPDH	*HORVU7Hr1G074690*	GTGAGGCTGGTGCTGATTACG	TGGTGCAGCTAGCATTTGAGAC
HvMADS2	*HORVU3Hr1G091000*	GCCCGAAGATACGAACCCTTCC	GGTGGTGCAAACTTACAGTGAGG
HvMADS2	*HORVU3Hr1G091000*	GTGTGTCCGATTTGATCTACTCC	CAAGATCCCCTCTATCCTGTATCG
HvMADS4	*HORVU1Hr1G063620*	ACACTCCACAGAGACAAGGG	GTTGGGAAACAACTAGCACTGG
HvMADS16	*HORVU7Hr1G091210*	CCCTCGTCCACTTTCTTCTCC	CGACACAAGGCAAGGTAAACG

Supplementary Table S5. PCR primer sequences for testing the presence of genes upstream and downstream of *HvMADS16* (bold) on chromosome 7H.

Gene ID	Forward primer	Reverse primer
HORVU7Hr1G091170	AGATATGAGATTGACAAGGTCACTGC	GGTAGAAGAACTAAGGTTCCACTTGC
HORVU7Hr1G091180	GACACTGAACTGAGCTGATGG	CAGGTGTGATACGAGTTGAAGG
HORVU7Hr1G091180	GAATGAGATGTTGTCGACTTGTCG	CACATTGTACTCCCTTCGTCTCG
HORVU7Hr1G091200	AGTAGAAAGGGGAAATTTAGTAGCG	TGAGCATGATGATGTTGAAGGAG
HORVU7Hr1G091210	**CCCTCGTCCACTTTCTTCTCC**	**CAATACACAGTCGAGCACTACG**
HORVU7Hr1G091220	GGAATCGGAGTAGACGCAAGC	GTGGCTAACGTCGATGGACC
HORVU7Hr1G091220	AAACCTTGGGTCGAGTAAAGCG	TGTTGGAACAGCACCTAACACC
HORVU7Hr1G091230	CAGTTATTGACAGACAGAGCTCC	GGTATGGGTACAGGATGTCATC
HORVU7Hr1G091240	GGCCATCAGGTCCTGTTTCAG	TTTCAGCTCCGTTGTAGTGTGG
HORVU7Hr1G091250	AGTGAGACAATCGACAGTAGCG	TGACAGTTGAGTGAGAGTGAGC

Supplementary Table S6. qRT-PCR primer sequences.

Gene name	Gene ID	Forward primer	Reverse primer	Acquisition Temperature (°C)
HvGAPDH	*HORVU7Hr1G074690*	GTGAGGCTGGTGCTGATTACG	TGGTGCAGCTAGCATTTGAGAC	82
HvCYCLO	*HORVU6Hr1G012570*	CCTGTCGTGTCGTCGGTCTAAA	ACGCAGATCCAGCAGCCTAAAG	81
HvTUB	*HORVU1Hr1G081280*	AGTGTCCTGTCCACCCACTC	AGCATGAAGTGGATCCTTGG	82
HvHSP70	*HORVU5Hr1G113180*	CGACCAGGGCAACCGCACCAC	ACGGTGTTGATGGGGTTCATG	85
HvMADS2	*HORVU3Hr1G091000*	CCAGCATGATATCGCCTTG	TCGAGCCAGTGGTGGATAA	82
*HvKinase**	*HORVU1Hr1G063610*	TTTGGCACCTTAGCCATCAT	ATGCCAAGATGTTCCTGGTC	76
*HvMADS4**	*HORVU1Hr1G063620*	ATGGAGCTCGGGTACCATC	CCTGCAGGTAGATGGAGCA	80
HvMADS16	*HORVU7Hr1G091210*	CCCAGGAGGCATACAAGAATCTGC	GCGGAAGGCGTACATGTCAGC	83
HvMADS3	*HORVU3Hr1G026650*	GCAGCAGCAGCATTACTCC	ACACATGCACGCGACAGTA	80
HvMADS58	*HORVU1Hr1G029220*	ATCATGCAGCAGCCTCAGT	GGTGTGGCCAAGCCTTAAT	77
HvMADS13	*HORVU1Hr1G023620*	TCAGCTGAACCTAGGCTGC	TTTGACAGGAATAGTTGAGTACTGGT	80
HvDL	*HORVU4Hr1G067780*	CCATGCAAGAGGCTGATGGACACG	GCGGCTGGTTCCTCCTGCAGTCAG	83
HvSL1	*HORVU3Hr1G003740*	GGAGGAGGAGGATTCAGGGGAGG	GCGTCGTGCTGTAGAGGTAGTGG	83

* Gene *HvMADS4* overlaps with *HvKinase*. *HvKinase* expression was also considered when assessing *HvMADS4* transcript abundance.

Supplementary Table S7. Primer sequences for *HvSL1* CRISPR knockout. gRNA sequence is underlined.

#	Gene ID	Forward primer	Reverse primer
gRNA1	*HORVU3Hr1G003740*	CTTGCTTGCCGTACTCCTCGTTTTAGAGCTAGAAAT	GAGGAGTACGGCAAGCAAGCAACACAAGCGGCAGC
gRNA2	*HORVU3Hr1G003740*	CCTGCACTCGTACACCTTGCGTTTTAGAGCTAGAAAT	GCAAGGTGTACGAGTGCAGGCGGCAGCCAAGCCAGCA

Supplementary Table S8. Primer sequence for cloning of *in situ hybridization* antisense (AS) and sense (S) probes. The T7 promoter sequence is underlined.

Gene name	Gene ID	Forward primer	Reverse primer
HvSL1 (AS)	*HORVU3Hr1G003740*	GCACCTTCCAAATCTCCATTGC	TAATACGACTCACTATAGGGTTGAGA CTTGCCGAACTTGAGG
HvSL1 (S)	*HORVU3Hr1G003740*	TAATACGACTCACTATAGGGGCACC TTCCAAATCTCCATTGC	TTGAGACTTGCCGAACTTGAGG
HvMADS2 (AS)	*HORVU3Hr1G091000*	CCTCAGTGCGGAGATTGATCG	TAATACGACTCACTATAGGGGCTGC AAAGTCCCTGTCTGG
HvMADS2 (S)	*HORVU3Hr1G091000*	TAATACGACTCACTATAGGGCCTCA GTGCGGAGATTGATCG	GCTGCAAAGTCCCTGTCTGG
HvMADS4 (AS)	*HORVU1Hr1G063620*	CTCAGGCATATGAAAGGCGAGG	TAATACGACTCACTATAGGGATGGA GCACCAGTTCAGACAGG
HvMADS4 (S)	*HORVU1Hr1G063620*	TAATACGACTCACTATAGGGCTCAG GCATATGAAAGGCGAGG	ATGGAGCACCAGTTCAGACAGG
HvMADS16 (AS)	*HORVU7Hr1G091210*	GCAAAGGATGGGTGAAGATCTGG	TAATACGACTCACTATAGGGGCGGA AGGCGTACATGTCAGC
HvMADS16 (S)	*HORVU7Hr1G091210*	TAATACGACTCACTATAGGGGCAAA GGATGGGTGAAGATCTGG	GCGGAAGGCGTACATGTCAGC
HvMADS3 (AS)	*HORVU3Hr1G026650*	AGGTTAACATGCAGCAGCAGC	TAATACGACTCACTATAGGGGGGAA GATATGCAACGCGATGG
HvMADS3 (S)	*HORVU3Hr1G026650*	TAATACGACTCACTATAGGGAGGTT AACATGCAGCAGCAGC	GGGAAGATATGCAACGCGATGG
HvMADS13 (AS)	*HORVU1Hr1G023620*	ATCAGGGCCAGGAAGAATGAGC	TAATACGACTCACTATAGGGCAGGTT GACTAGAACTGATGAGCC
HvMADS13 (S)	*HORVU1Hr1G023620*	TAATACGACTCACTATAGGGATCAG GGCCAGGAAGAATGAGC	CAGGTTGACTAGAACTGATGAGCC
HvDL (AS)	*HORVU4Hr1G067780*	TTCCATGCAAGAGGCTGATGG	TAATACGACTCACTATAGGGGCTTTG ATACGCTGTATTTCCTCC
HvDL (S)	*HORVU4Hr1G067780*	TAATACGACTCACTATAGGGTTCCAT GCAAGAGGCTGATGG	GCTTTGATACGCTGTATTTCCTCC

Supplementary Table S9. Primer sequences for BiFC cloning. HindIII restriction site is underlined in all forward primers, XmaI restriction site is underlined in all reverse primers.

Gene name	Gene ID	Forward primer	Reverse primer
HvMADS2	*HORVU3Hr1G091000*	TTCAAGCTTACATGGGGCGCGGGAAG ATCG	ACCCGGGATCTAGGTGTCCTCCTGCA GATTGGG
HvMADS4	*HORVU1Hr1G063620*	TTCAAGCTTACATGGGGCGCGGCAAG ATCG	ACCCGGGATCTACTTGTCTTCCTGCA AGTTGGGGTG
HvMADS16	*HORVU7Hr1G091210*	TTCAAGCTTACATGGGGCGGGGGAAG ATCG	ACCCGGGTATTATCCGAGGCGCAGGT CGTG
△HvMADS16	*HORVU7Hr1G091210*	TTCAAGCTTACATGGGGCGGGGGAAG ATCG	ACCCGGGTACTTGATGTCGGTGCCGG TGC

Supplementary Table S10. Variation in floral organ frequency in wild-type and *mov2.g*. As an example, phenotypic variation is reported for six individual *mov2.g* florets from different spikes. All *mov2.g* florets contained 2 unaltered lodicules. WT indicates a wild-type barley floret.

Floret #	Stamen	Carpel/ carpel-like	Partially converted stamen	Comments
WT	3	1	0	
mov2.g	0	4	0	At least 2 carpels appear to contain ovules
mov2.g	0	5	0	
mov2.g	0	1	3	Three carpelloid/partially converted stamens
mov2.g	0	3	1	
mov2.g	0	6	0	Carpel-like organs vary in shape and size
mov2.g	0	7	0	Several exposed ovule-like structures

Supplementary Table S11. Annotated genes present in the mapped *mov2* critical interval between flanking markers chr3H_9748112 and chr3H_10289104. Annotations and genomic coordinates are based on the Morex reference assembly Hv_IBSC_PGSB_v2, gene order is based on Morex Scaffold_1432 (Dr. Martin Mascher, IPK Gatersleben, Germany). *HvSL1* (*HORVU3Hr1G003740*) is indicated in bold, genes tested by PCR are indicated with *.

Gene	Start	End	Strand	Annotation
*HORVU3Hr1G003690 **	9,737,630	9,749,764	+	N.A.
HORVU3Hr1G003700	9,753,370	9,754,129	-	Undescribed protein
HORVU3Hr1G003710	9,756,062	9,756,300	-	Undescribed protein
HORVU3Hr1G003720	9,757,456	9,764,144	-	Unknown function
HORVU3Hr1G003730	9,774,072	9,774,466	-	Undescribed protein
*HORVU3Hr1G003760 **	9,942,084	9,945,779	+	Protein of unknown function (DUF1666)
HORVU3Hr1G003750	9,937,908	9,938,930	-	Undescribed protein
HORVU3Hr1G003740 *	**9,908,524**	**9,910,297**	**-**	**Zinc finger protein 6**
HORVU3Hr1G003780	10,031,768	10,032,424	-	Undescribed protein
HORVU3Hr1G003770	10,030,637	10,031,653	-	Undescribed protein
HORVU3Hr1G003800	10,168,437	10,169,618	-	Undescribed protein
*HORVU3Hr1G003810 **	10,169,902	10,172,670	+	Disease resistance protein
*HORVU3Hr1G003820 **	10,173,750	10,180,522	+	Synaptotagmin A
HORVU3Hr1G003830	10,180,811	10,181,062	-	Undescribed protein
HORVU3Hr1G003840	10,200,846	10,209,364	+	BnaA07g10090D protein
HORVU3Hr1G003850	10,208,187	10,213,828	-	Nuclease S1
HORVU3Hr1G003870	10,259,028	10,259,724	+	Endonuclease 2
HORVU3Hr1G003790	10,058,110	10,058,378	+	Undescribed protein
HORVU3Hr1G003860	10,224,976	10,227,068	+	Endonuclease 2
HORVU3Hr1G003880	10,288,324	10,289,509	-	Unknown function

Supplementary Table S12. BLASTp results using barley HvSL1 as query against the rice genome (RGAP 7). All hits are shown.

Gene ID	E-value	Query coverage (%)	Sequence identity (%)
LOC_Os01g03840 (OsSL1)	6e-88	99.25	65.4
LOC_Os01g32920	1e-06	14.98	52.5

Supplementary Table S13. BLASTp results using rice OsSL1 as query against the barley genome (Hv_IBSC_PGSB_v2). Only the top five hits are shown.

Gene ID	E-value	Query coverage (%)	Sequence identity (%)
HORVU3Hr1G003740 (HvSL1)	3.2e-87	97	65.0
HORVU3Hr1G007060	4.2e-07	11	54.8
HORVU5Hr1G103440	8e-07	13	51.4
HORVU4Hr1G085640	8e-07	22	59.4
HORVU5Hr1G018830	1.1e-06	16	62.1

Supplementary Table S14. Observed segregation ratios of *mov1* phenotype in heterozygote growing material.

Group #	Observed *mov1* phenotype	Observed wild-type phenotype	Total	χ^2 value	P-value	Output
1	68 (24%)	214 (76%)	282	0.118	0.731	**ACCEPT H_0**
2	10 (25%)	30 (75%)	40	0.000	1.000	**ACCEPT H_0**
3	10 (26%)	29 (74%)	39	0.009	0.926	**ACCEPT H_0**
4	9 (19%)	39 (81%)	48	1.000	0.317	**ACCEPT H_0**
5	41 (24%)	133 (76%)	174	0.192	0.662	**ACCEPT H_0**

H_0 = The observed phenotypes segregate with a 3:1 ratio

H_1 = Not H_0; observed phenotypes segregate differently from 3:1 ratio

Degrees of freedom (DF) = 1

Supplementary Table S15. Genes on chromosome 7H tested by PCR. All genes were present in *mov1*, except for genes shown in bold. Annotations and genomic coordinates based on the Morex reference assembly Hv_IBSC_PGSB_v2. Presence of gene *HORVU7Hr1G091190* could not be tested due to sequence repetitiveness and is therefore considered within the deletion.

Gene	Start	End	Strand	Annotation
HORVU7Hr1G091170	556,426,170	556,428,917	+	Protein FMP32, mitochondrial
HORVU7Hr1G091180	556,432,734	556,434,307	+	B-box zinc finger family protein
HORVU7Hr1G091190	**557,017,373**	**557,018,262**	**-**	**40S ribosomal protein**
HORVU7Hr1G091200	**557,181,329**	**557,182,073**	**-**	**Undescribed protein**
HORVU7Hr1G091210	**557,244,345**	**557,245,839**	**-**	**MADS-box transcription factor 16**
HORVU7Hr1G091220	557,387,804	557,392,562	+	UPF0183 protein
HORVU7Hr1G091230	557,392,887	557,396,669	+	Unknown protein
HORVU7Hr1G091240	557,397,068	557,398,785	-	Alpha-amylase-like
HORVU7Hr1G091250	557,426,822	557,429,314	-	Alpha-amylase-like

Supplementary Fig. S1. Histological sections of *mov2.g* carpels. Transverse sections of mature (**A**) wild-type and (**B**) *mov2.g* carpels stained with toluidine blue. Inset in (**A**) shows characteristics of a wild-type female gametophyte. Red arrows indicate: (**I**) synergid cells, (**II**) egg cell, (**III**) central cell and (**IV**) antipodal cells in serial sections. Black asterisks indicate ovules, whereas black arrows indicate additional carpel-like structures. Scale bars: 100 µm.

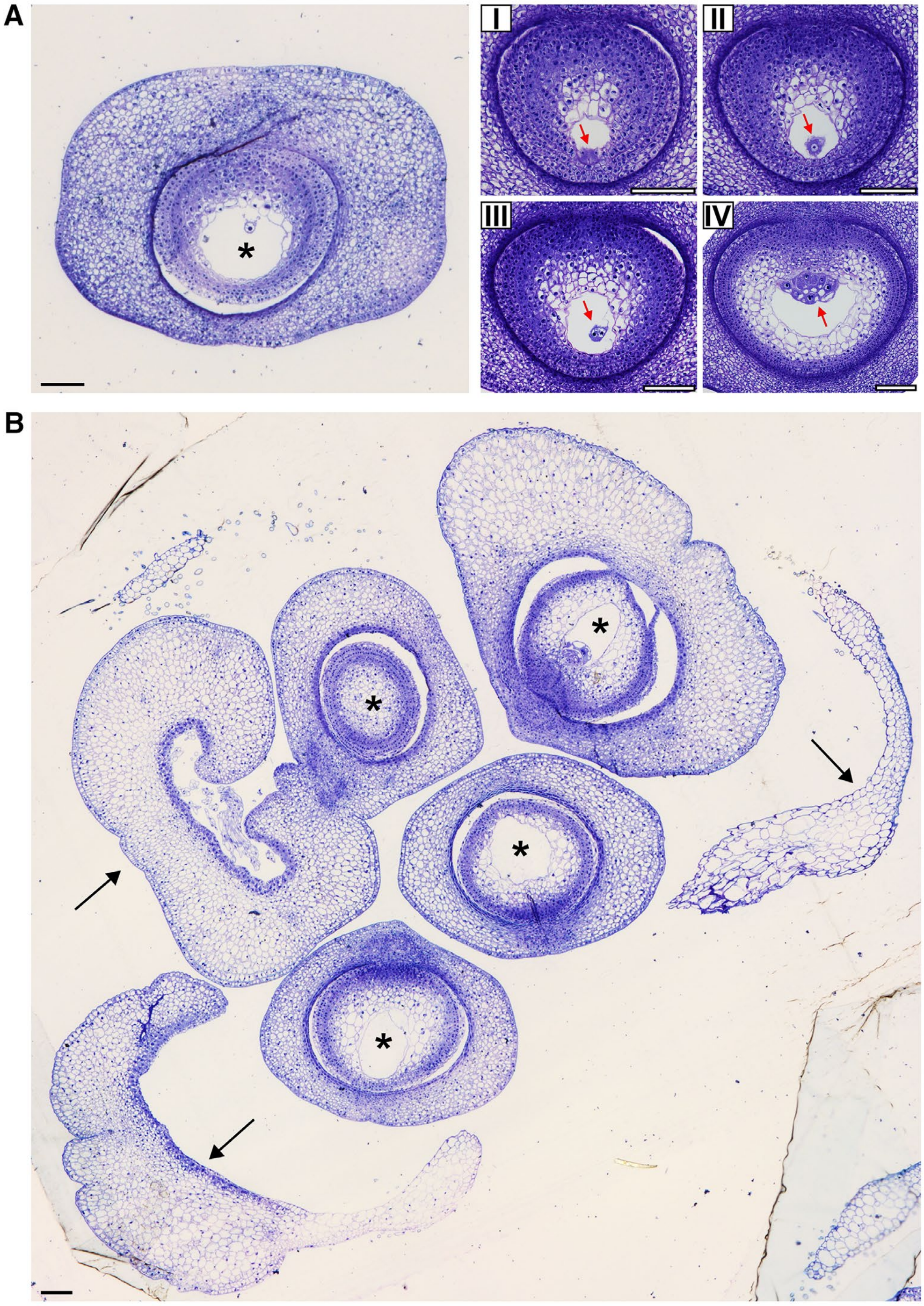

Supplementary Fig. S2. Spike morphology in wild-type and *mov2.g* plants. Representative images for (**A**) wild type and (**B-D**) *mov2.g* plants. Scale bars: 1 cm.

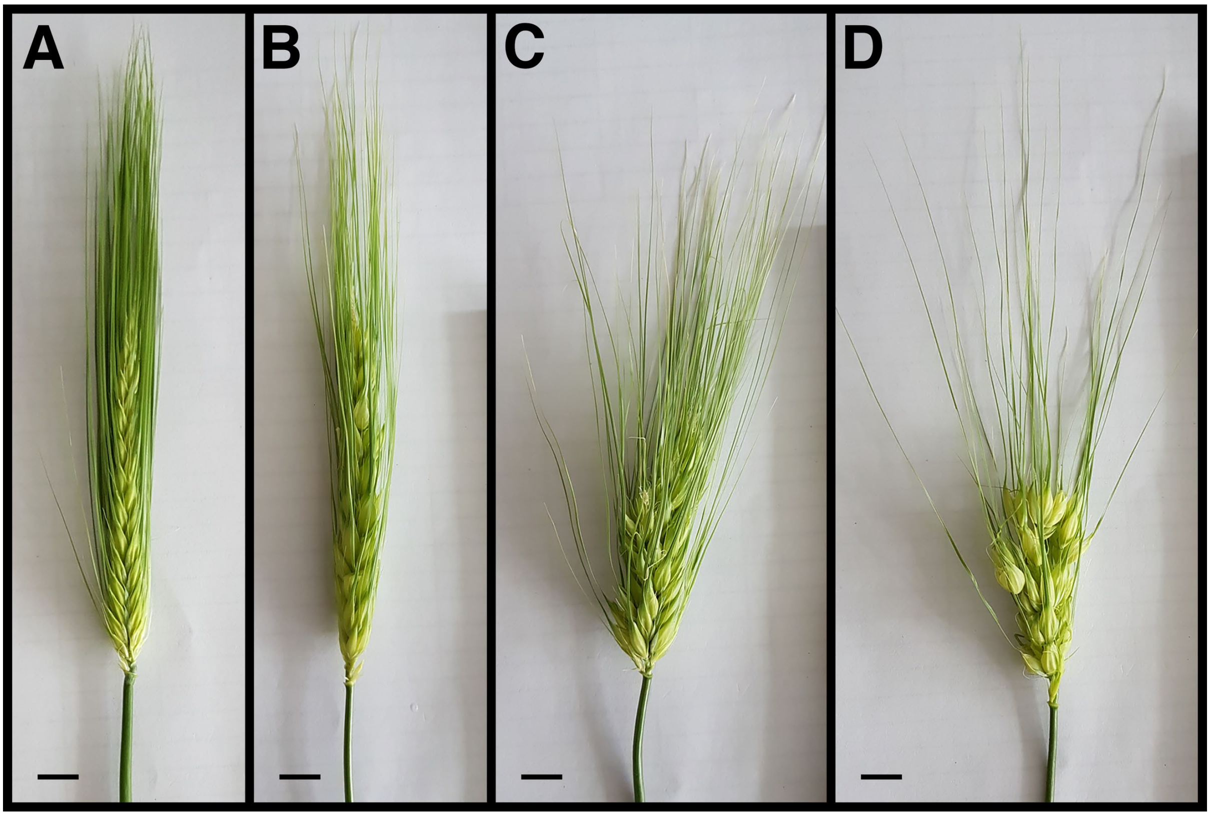

Supplementary Fig. S3. Mapping of the *mov2* locus in a *mov2.g* x Morex bi-parental population. *mov2* was initially mapped to a ~1.9 Mb interval between markers chr3H_9095799 and chr3H_11039299 based on 352 F_2 segregants. Fine mapping using 179 F_3 recombinants reduced the critical interval to ~449 Kb between markers chr3H_9748112 and chr3H_10289104. Markers are represented by vertical black lines along the chromosome. Marker order is based on the genetic map and recombination frequency (%) is reported at the bottom for each marker. Examples of mapping in F_3 recombinants: line names are indicated on the side, lines exhibiting the *mov2.g* phenotype are indicated in bold. Green: *mov2.g* allele; grey: Morex allele.

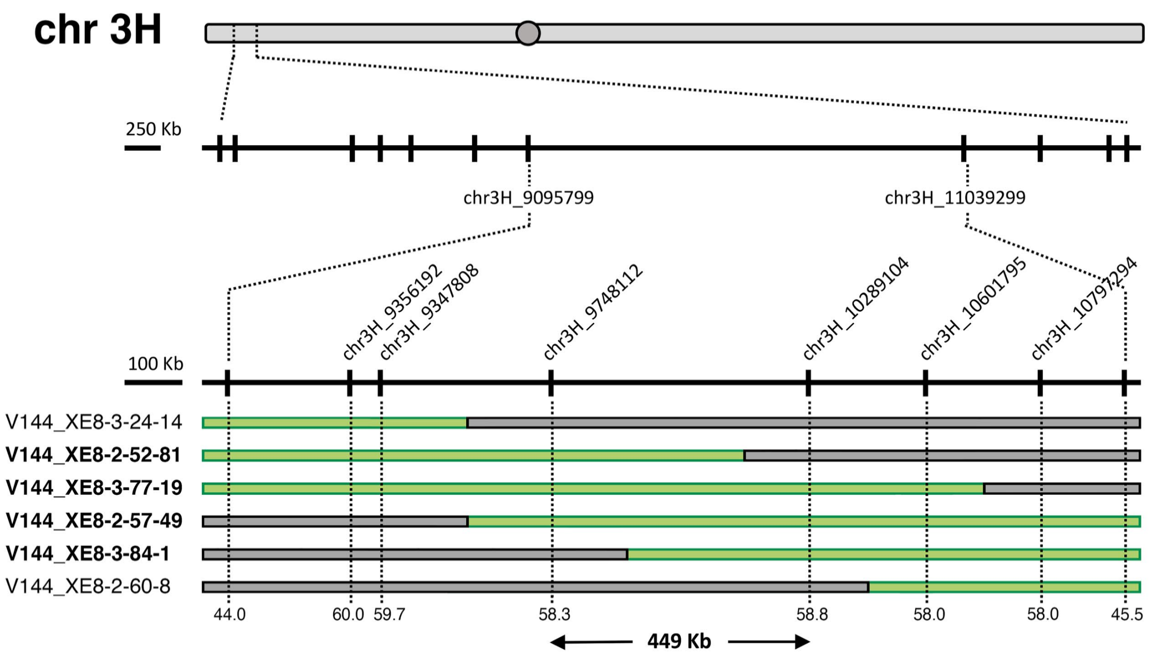

Supplementary Fig. S4. Heatmap of gene expression for genes in the critical *mov2* interval. Heatmap shows RNAseq values (RPKM) in different plant tissues for genes present in the mapped interval. Gene order is based on Morex Scaffold_1432 (Dr. Martin Mascher, IPK Gatersleben, Germany). *HvSL1* (*HORVU3Hr1G003740*) is indicated in bold and with red box. For each tissue, development based on the Zadoks or Waddington growth scale is provided.

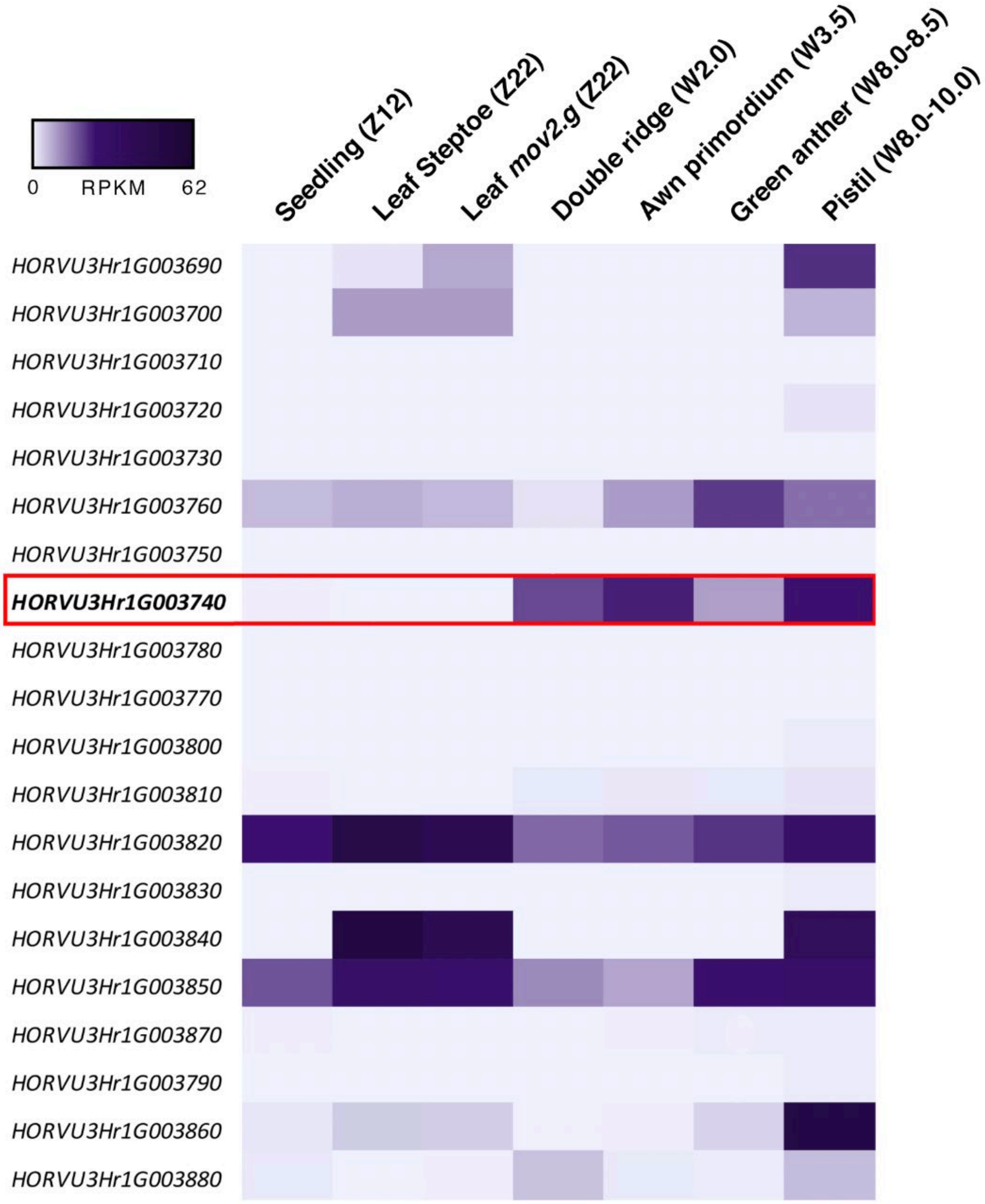

Supplementary Fig. S5. *HvSL1* and *OsSL1* gene models and alignment. (**A**) Schematic representation of barley (*HvSL1*) and rice (*OsSL1*) gene models. For each gene, the length in base pairs of Untranslated Regions (UTR – white boxes), protein-coding regions (black boxes) and introns (solid line) is indicated. (**B**) Protein alignment of barley (HvSL1) and rice (OsSL1).

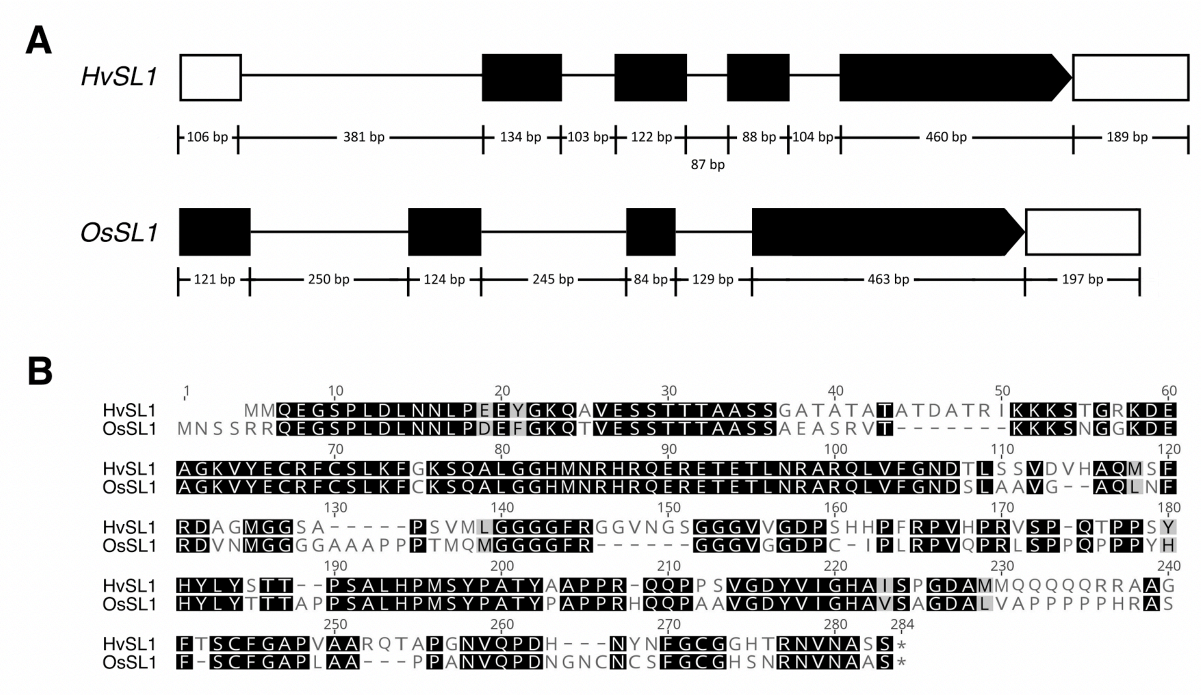

Supplementary Fig. S6. *HvSL1* deletion in *mov2.g*. (**A**) *HvSL1* appears to be absent in *mov2.g* plants when assayed by PCR. (**B**) *HvSL1* is expressed in wild-type (solid black line), but not in *mov2.g* (dashed black line) developing inflorescences at stages W2.0 (double ridge), W3.5 (stamen primordia), W4.5 (carpel primordium) and W6.0 (stamen and carpel development). Error bars represent ± Standard Error. For each timepoint, two-tailed T-test P-values ≤0.05 (*), ≤0.005 (**) and ≤0.001 (***) are shown for differences between wild type and *mov2.g*. For each sample n = 3 independent biological replicates.

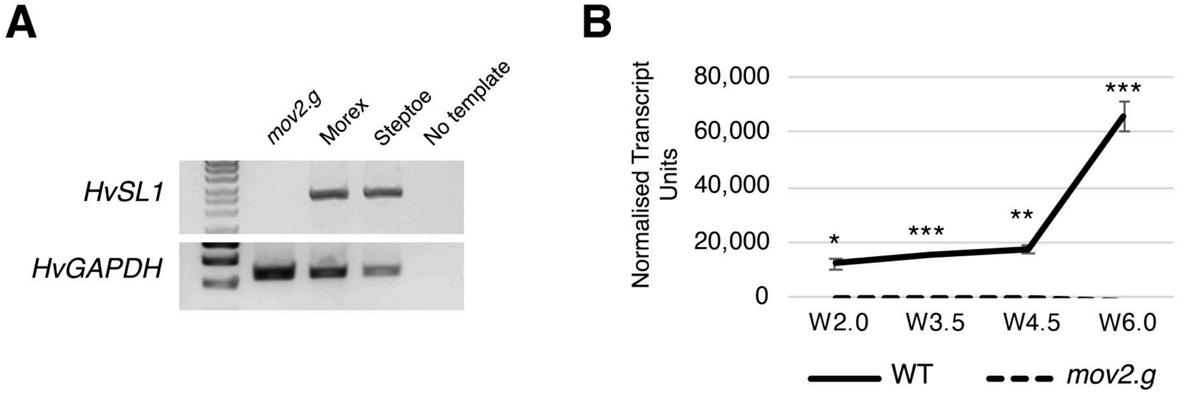

Supplementary Fig. S7. CRISPR design and analysis of *Hvsl1*-knockout plants. (**A**) Schematic representation of the *HvSL1* gene structure showing the position of gRNA1 and gRNA2 in red, protein-coding regions as grey boxes and UTR regions as white boxes. (**B**) Edits detected in gRNA1 (left column) and gRNA2 (right column) in CRISPR *Hvsl1*-knockout plants with relative insertions/deletions shown in red and indicated in brackets. PAM sequence is underlined.

A

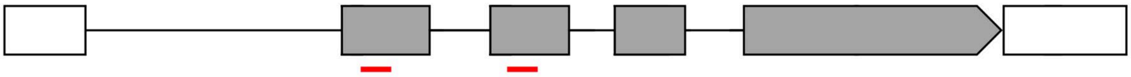

B

Line		gRNA1		gRNA2
	WT:	GCTTGCTTGCCGTACTC–CTCCGG	WT:	CCTGCACTCGTACACCTTGCCGG
HvSL1-1	(+1)	GCTTGCTTGCCGTACTCTCTC		
	(-1)	GCTTGCTTGCCGTACT––CTC		
	WT:	GCTTGCTTGCCGTACTCCTCCGG	WT:	CCTGCACTCGTACACCTTGCCGG
HvSL1-3	(-1)	GCTTGCTTGCCGTACT–CTC		
	(+89/-6)	GGCTCAAGTCTCCAACAACT...		
	WT:	GCTTGCTTGCCGTACTC–CTCCGG	WT:	CCTGCACTCGTACACCTTGCCGG
HvSL1-5	(+1)	GCTTGCTTGCCGTACTCTCTC		
	(-1)	GCTTGCTTGCCGTACT––CTC		
	WT:	GCTTGCTTGCCGTACTCCTCCGG	WT:	CCTGCACTCGTACACCTTGCCGG
HvSL1-6	(-1)	GCTTGCTTGCCGTACT–CTC		
	WT:	GCTTGCTTGCCGTACTC–CTCCGG	WT:	CCTGCACTCGTACACCTTGCCGG
HvSL1-10	(+1)	GCTTGCTTGCCGTACTCTCTC		
	(-1)	GCTTGCTTGCCGTACT––CTC		
	(-2)	GCTTGCTTGCCGTAC–––CTC		

Supplementary Fig. S8. BiFC assays showing interaction between barley B-class genes. nYFP indicates N-terminal of YFP (1-174), while cYFP indicates C-terminal split of YFP (175-241). Scale bars: 200 μm. A previously published interaction between rice proteins OsERS1 and OsARC was used as positive control (Yang *et al*., 2018). For each interaction, n = 4 independent transfections.

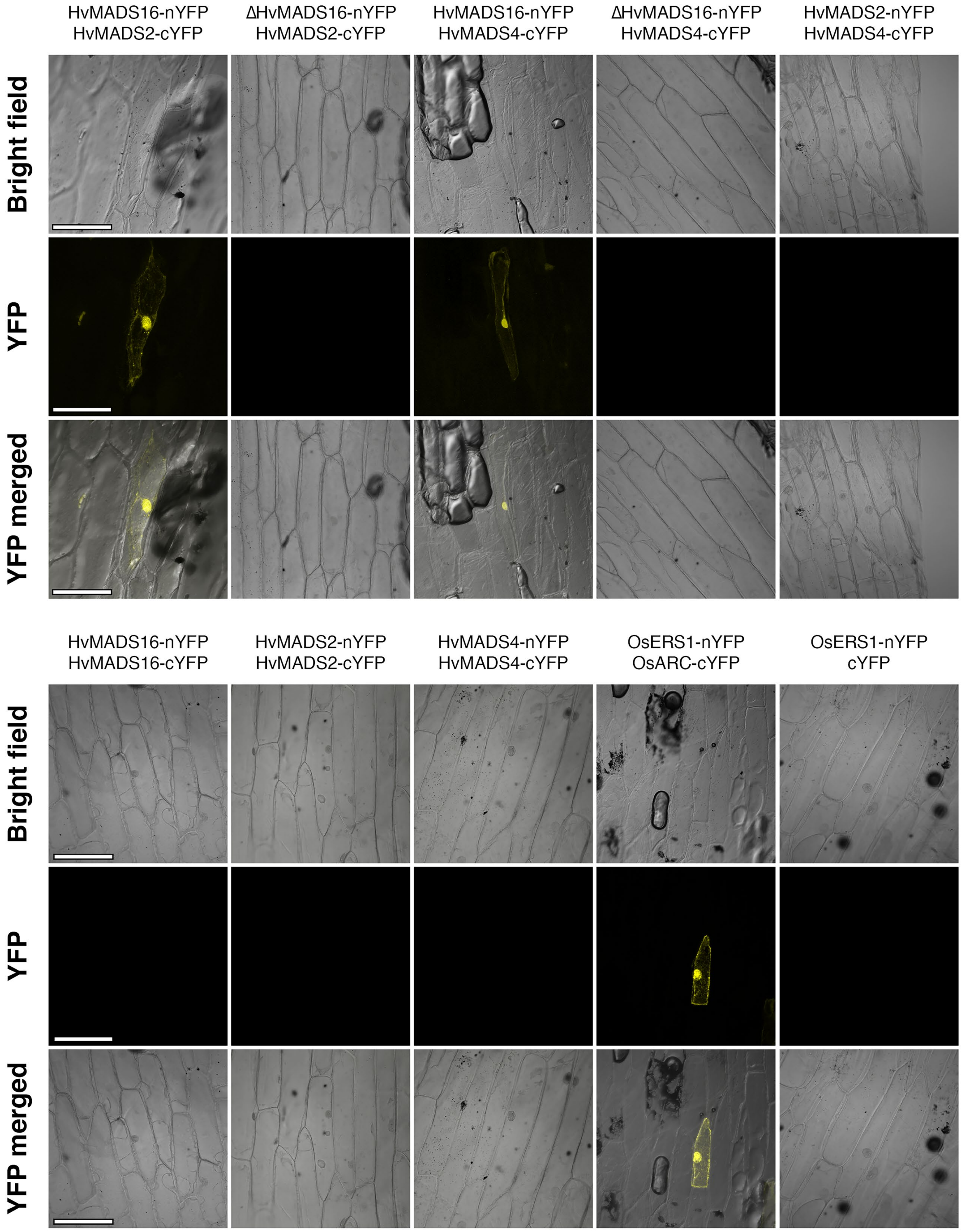

Supplementary Fig. S9. Transfection efficiency and transcript abundance in barley protoplasts. (**A**) Efficiency of protoplast transfection was calculated by averaging the number of protoplasts expressing YFP in three representative images. Scale bars: 100 µm. (**B**) *HvSL1* expression as assayed by qRT-PCR in protoplasts transfected with mock or with a construct driving constitutive *HvSL1* expression. Constitutive *HvSL1* expression is driven by the Cauliflower Mosaic Virus 35S promoter. Fold change is reported relative to barley glyceraldehyde 3-phosphate dehydrogenase (*HvGAPDH*) and cyclophilin (*HvCYCLO*). (**C**) Expression of endogenous B-class genes *HvMADS2*, *HvMADS4* and *HvMADS16* in protoplasts transfected with mock or with a construct driving constitutive *HvSL1* expression. Fold change is reported relative to the mock condition. Two-tailed T-test P-value ≤0.005 (**) is shown for differences between treatments, ns indicates no significant difference. For each sample n = 3 independent biological replicates.

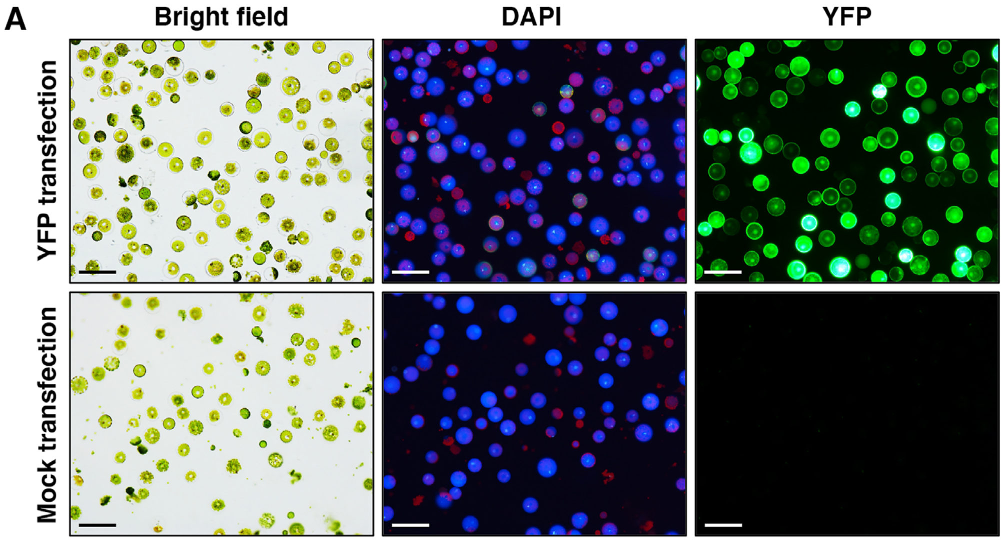

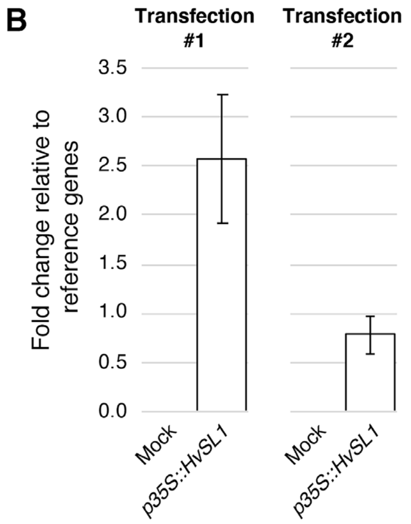

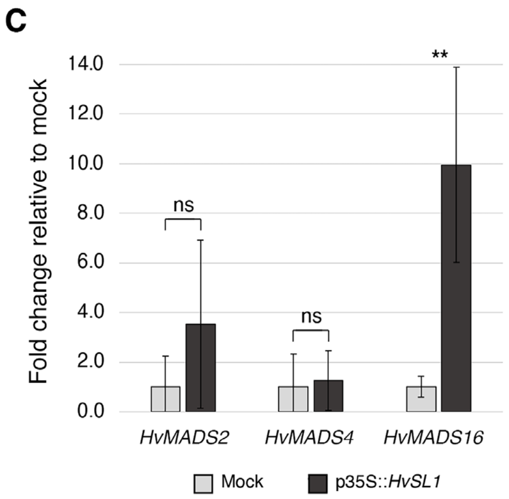

Supplementary Fig. S10. Histological sections of *mov1* carpels. Transverse sections of mature (**A**) wild-type and (**B and C**) *mov1* carpels stained with toluidine blue. Black asterisks indicate the ovule-like structures in the multiovary mutant. Scale bars: 100 μm.

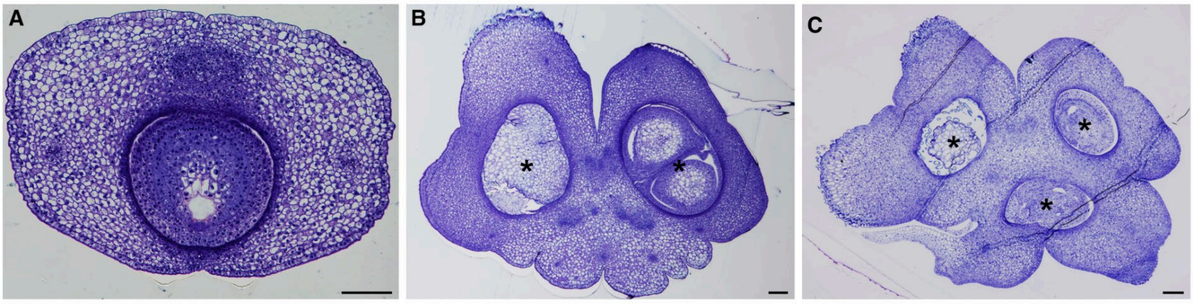

Supplementary Fig. S11. Details of *mov1* inflorescence development. Enlargement of the *mov1* inflorescence shown in Fig. 7 at W5.0, highlighting in greater detail the multiple florets (numbered 1 & 2) arising from a single floral meristem. Scale bars: 500 μm.

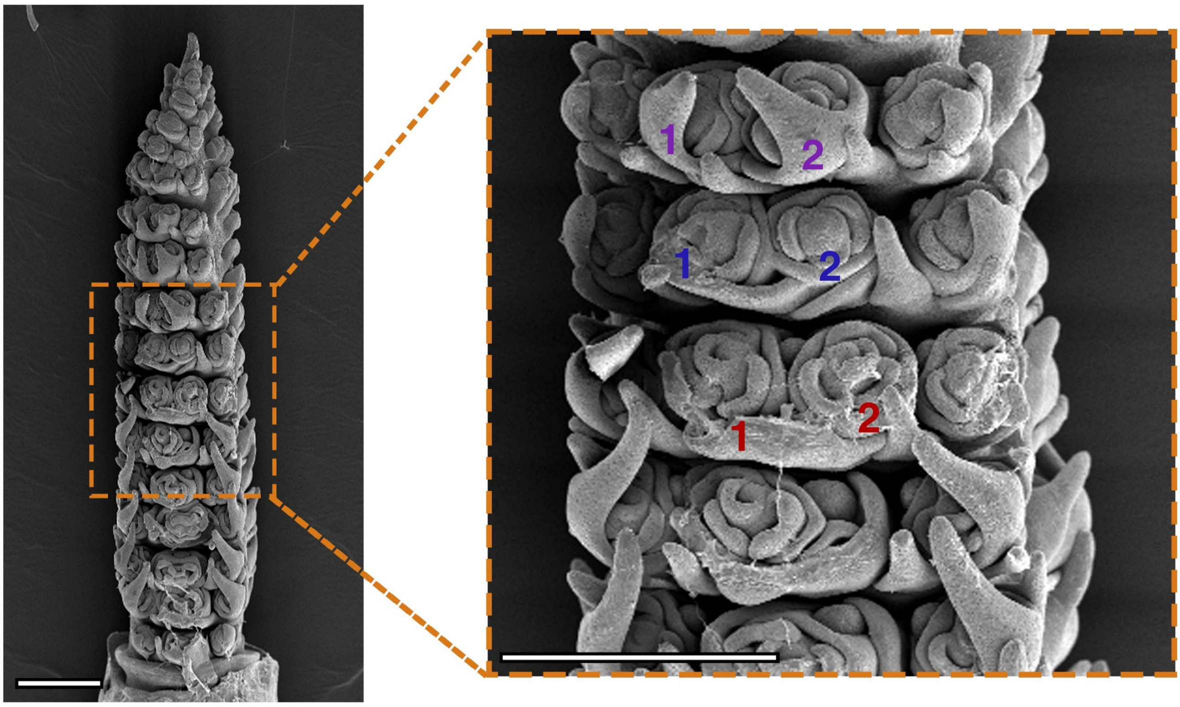

Supplementary Fig. S12. Characterization of the *mov1* deletion and *HvMADS16*. (**A**) *HvMADS16* is the only B-class gene physically absent in *mov1* when assayed by PCR. The barley glyceraldehyde 3-phosphate dehydrogenase *HvGAPDH* gene was used as reference. (**B**) Transcript abundance of *HvMADS16* in a Steptoe tissue series as assayed by qRT-PCR. For details about tissue sampling refer to Materials and Methods. (**C**) Schematic representation of the deletion in *mov1* as assayed by PCR. Deletion size in *mov1* is estimated to be no bigger than 0.95 Mb, based on the Morex reference assembly Hv_IBSC_PGSB_v2. (**D**) *HvMADS16* gene structure; length in base pairs of Untranslated Regions (UTR), protein-coding regions (black) and introns (solid line) is indicated. (**E**) Protein alignment of barley (HvMADS16) and rice (OsMADS16), sharing 88.3% sequence identity.

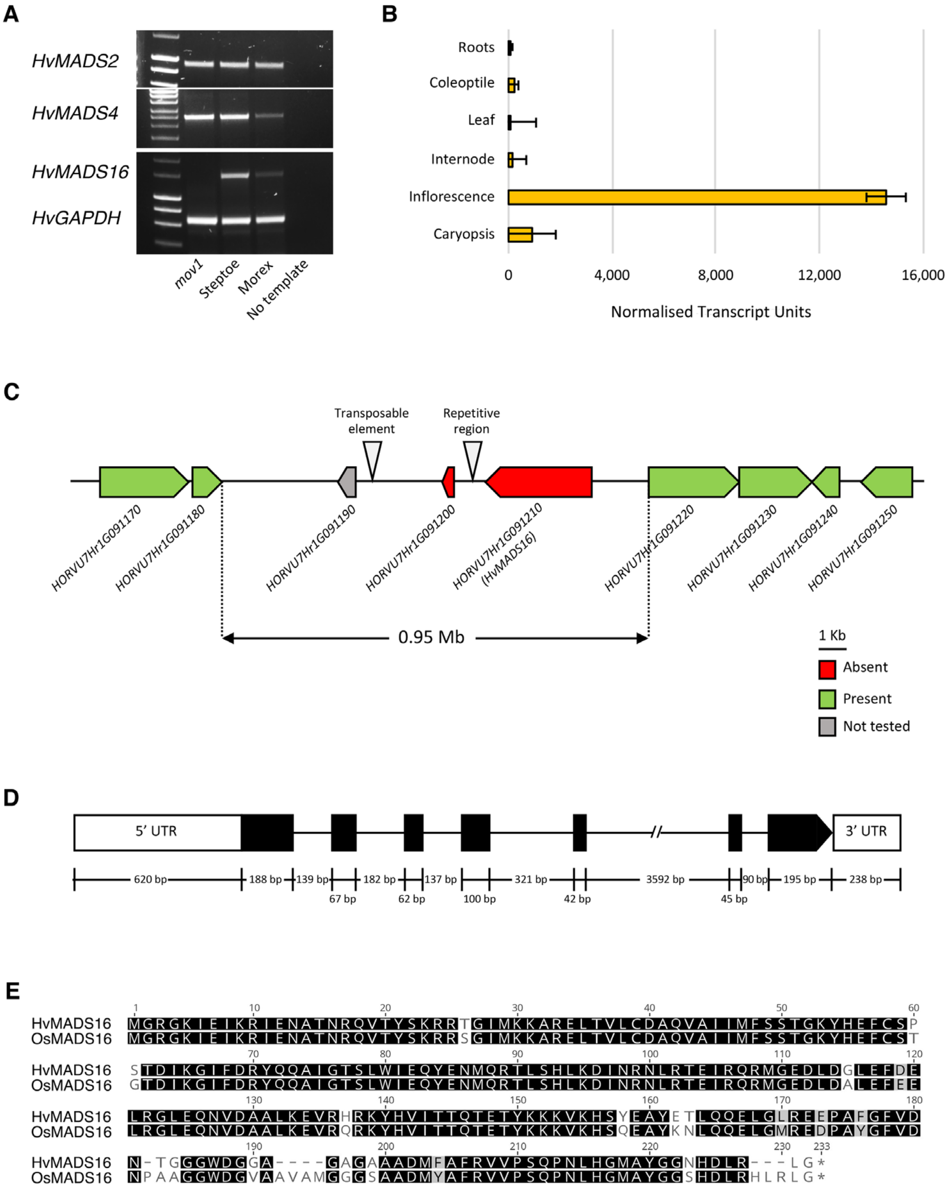

Supplementary Fig. S13. *In situ* hybridization with sense probes on wild-type and *mov1* inflorescences. Sense probes for *HvMADS16*, *HvMADS3*, *HvMADS13* and *HvDL* were assayed on wildtype (WT) and *mov1* inflorescences at stage W6.0. Lemma (le), lodicule (lo), stamen (st), carpel (ca), ovule (ov) and carpel-like structure (cl). Scale bars: 250 µm.

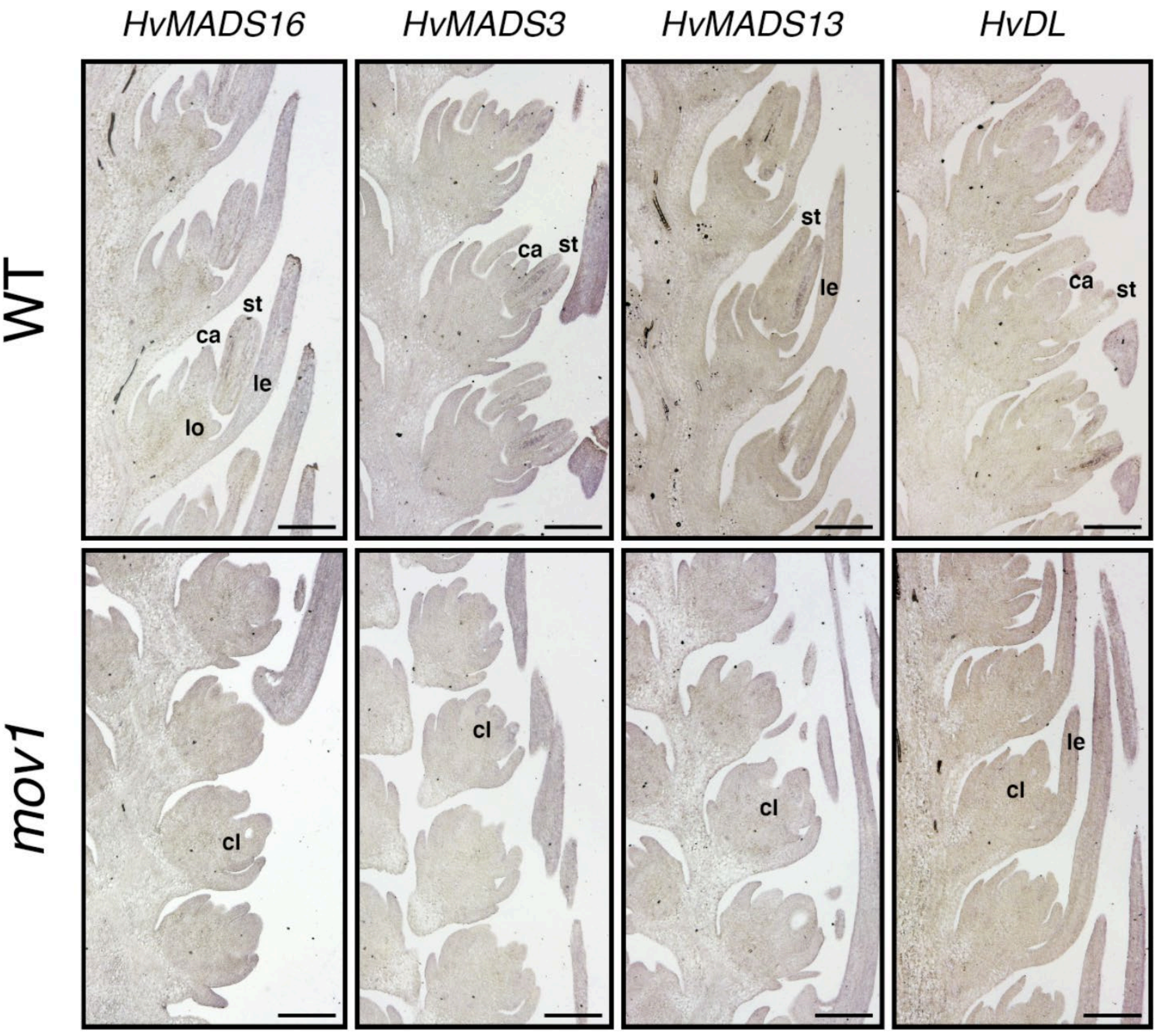

Supplementary Information References

Yang X, Li G, Tian Y, Song Y, Liang W, Zhang D. 2018. A rice glutamyl-tRNA synthetase modulates early anther cell division and patterning. Plant Physiology. 177,728–744.
